# Novel Synthetic Coumarin-Chalcone Derivative (E)-3-(3-(4-(Dimethylamino)Phenyl)Acryloyl)-4-Hydroxy-2*H*-Chromen-2-One Activates CREB-Mediated Neuroprotection in A*β* and Tau Cell Models of Alzheimer's Disease

**DOI:** 10.1155/2021/3058861

**Published:** 2021-11-13

**Authors:** Ya-Jen Chiu, Te-Hsien Lin, Chiung-Mei Chen, Chih-Hsin Lin, Yu-Shan Teng, Chung-Yin Lin, Ying-Chieh Sun, Hsiu Mei Hsieh-Li, Ming-Tsan Su, Guey-Jen Lee-Chen, Wenwei Lin, Kuo-Hsuan Chang

**Affiliations:** ^1^Department of Life Science, National Taiwan Normal University, Taipei 11677, Taiwan; ^2^Department of Neurology, Chang Gung Memorial Hospital, Chang Gung University School of Medicine, Taoyuan 33302, Taiwan; ^3^Medical Imaging Research Center, Institute for Radiological Research, Chang Gung University/Chang Gung Memorial Hospital, Taoyuan 33302, Taiwan; ^4^Department of Chemistry, National Taiwan Normal University, Taipei 11677, Taiwan

## Abstract

Abnormal accumulations of misfolded A*β* and tau proteins are major components of the hallmark plaques and neurofibrillary tangles in the brains of Alzheimer's disease (AD) patients. These abnormal protein deposits cause neurodegeneration through a number of proposed mechanisms, including downregulation of the cAMP-response-element (CRE) binding protein 1 (CREB) signaling pathway. Using CRE-GFP reporter cells, we investigated the effects of three coumarin-chalcone derivatives synthesized in our lab on CREB-mediated gene expression. A*β*-GFP- and *Δ*K280 tau_RD_-DsRed-expressing SH-SY5Y cells were used to evaluate these agents for possible antiaggregative, antioxidative, and neuroprotective effects. Blood-brain barrier (BBB) penetration was assessed by pharmacokinetic studies in mice. Of the three tested compounds, (E)-3-(3-(4-(dimethylamino)phenyl)acryloyl)-4-hydroxy-2*H*-chromen-2-one (LM-021) was observed to increase CREB-mediated gene expression through protein kinase A (PKA), Ca^2+^/calmodulin-dependent protein kinase II (CaMKII), and extracellular signal-regulated kinase (ERK) in CRE-GFP reporter cells. LM-021 exhibited antiaggregative, antioxidative, and neuroprotective effects mediated by the upregulation of CREB phosphorylation and its downstream brain-derived neurotrophic factor and BCL2 apoptosis regulator genes in A*β*-GFP- and *Δ*K280 tau_RD_-DsRed-expressing SH-SY5Y cells. Blockage of the PKA, CaMKII, or ERK pathway counteracted the beneficial effects of LM-021. LM-021 also exhibited good BBB penetration ability, with brain to plasma ratio of 5.3%, in *in vivo* pharmacokinetic assessment. Our results indicate that LM-021 works as a CREB enhancer to reduce A*β* and tau aggregation and provide neuroprotection. These findings suggest the therapeutic potential of LM-021 in treating AD.

## 1. Introduction

Alzheimer's disease (AD) is a progressive and irreversible neurodegenerative disease that gradually impairs memory and cognitive function [1]. The pathological hallmarks of AD include deposits of the extracellular senile plaques and intracellular neurofibrillary tangles, which eventually lead to extensive loss of neurons and synapses and brain atrophy. Senile plaques are caused by the aggregation and deposition of amyloid *β* peptides (A*β*) [2], fragments of the amyloid peptide precursor protein (APP) that result from its cleavage by *β*- and *γ*-secretases [3]. A*β* tends to form oligomers and other high-order polymerized structures that are toxic to neurons [4]. Neurofibrillary tangles are composed of abnormally hyperphosphorylated microtubule-associated protein tau (MAPT) [5, 6], which are also involved in the neurodegenerative process [7]. Accumulation of A*β* is considered to be an early event and could trigger or accelerate tau pathology in AD [8].

The molecular mechanisms underlying AD aetiology and pathogenesis remain elusive. Compelling evidence suggests that changes in cAMP-response-element (CRE) binding protein 1- (CREB-) mediated gene expression play an important role in AD pathogenesis. CREB is a critical nuclear transcription factor enhancing cognition, memory formation, and neuronal survival [9]. CREB activation occurs primarily through the phosphorylation of serine 133 by either of several kinases: protein kinase A (PKA) [10], Ca^2+^/calmodulin-dependent protein kinase II (CaMKII) [11], extracellular signal-regulated kinase (ERK) [12], and phosphatidylinositol 3-kinase (PI3K) [13]. Phosphorylated CREB binds to the coactivators CREB binding protein (CBP) and E1A binding protein p300 (EP300), resulting in the expression of target genes [14]. CREB expression is downregulated in hippocampal neurons of *APP*-transgenic mice, hippocampal tissues of AD patients, and A*β*-treated rat hippocampal neurons [15]. In cultured neurons, A*β* interferes with CREB activation and expression of its downstream genes [16]. The A*β*-impaired hippocampal synaptic plasticity and long-term potentiation can be reversed by activating the CREB signaling pathway [17]. These observations provide important insights into the role of CREB signaling in the development of AD.

At present, no treatment is available to slow the neurodegeneration of AD. The central role of the CREB signaling pathway in AD neurodegeneration suggests its potential as a therapeutic target for AD. The cognitive function of presenilin 1 (*PS1*)/*APP* transgenic mice is significantly improved by increasing CREB phosphorylation with phosphodiesterase inhibitors [18, 19]. In our previous study, a synthetic coumarin-chalcone hybrid LM-031 upregulates CREB and demonstrates the neuroprotective potential to reduce A*β*, tau, expanded polyglutamine (polyQ) aggregation, and oxidative stress [20–22]. Here, we investigate three synthetic coumarin-chalcone derivatives made in our laboratory to determine their effects on CREB expression. The neuroprotective potential of these compounds for treating AD was further evaluated using A*β*-GFP- and *Δ*K280 tau_RD_-DsRed-expressing SH-SY5Y cells [23, 24].

## 2. Materials and Methods

### 2.1. Test Compounds

The coumarin-chalcone derivatives (E)-4-hydroxy-3-(3-(*p*-tolyl)acryloyl)-2*H*-chromen-2-one (LM-016), (E)-3-(3-(4-(dimethylamino)phenyl)acryloyl)-4-hydroxy-2*H*-chromen-2-one (LM-021), and (E)-3-(3-(furan-2-yl)acryloyl)-4-hydroxy-2*H*-chromen-2-one (LM-022) were synthesized in our laboratory as previously described and examined by using NMR spectroscopy [25, 26]. The LM-016, LM-021, and LM-022 stayed soluble in cell culture medium at concentrations up to 100 *μ*M. Curcumin, Congo red, forskolin (positive controls for A*β*-GFP, ∆K280 tau_RD_ [tau repeat domain]-DsRed, and CRE-GFP fluorescence assays), and kaempferol (an antioxidant used as a positive control in radical-scavenging assay) were obtained from Sigma-Aldrich Co. (St. Louis, MO, USA).

### 2.2. Bioavailability and Blood-Brain Barrier Permeation Prediction

To calculate the molecular weight (MW), hydrogen bond donor (HBD), hydrogen bond acceptor (HBA), octanol-water partition coefficient (cLogP), and polar surface area (PSA) of the coumarin-chalcone derivatives, ChemDraw (http://www.perkinelmer.com/tw/category/chemdraw) was applied. In addition, scores of blood-brain barrier (BBB) permeation were evaluated using an online BBB predictor (https://www.cbligand.org/BBB/).

### 2.3. 1,1-Diphenyl-2-Picrylhydrazyl Assay

Stable free radical 1,1-diphenyl-2-picrylhydrazyl (DPPH) (Sigma-Aldrich) was applied to test the potential radical-scavenging activity of the coumarin-chalcone derivatives [27]. In brief, the compounds (10–160 *μ*M) were added to an ethanol solution containing 100 *μ*M DPPH. The mixture was vortexed for 15 sec, left to stand for 30 min at room temperature, and scavenging capacity was measured at a wavelength of 517 nm using a Multiskan GO microplate spectrophotometer (Thermo Fisher Scientific, Waltham, MA, USA). The formula 1 − (absorbance of sample/absorbance of control) × 100% was used to compute the radical-scavenging activity, and the interpolation method was used to calculate the concentration of 50% of maximal effect (EC_50_).

### 2.4. Thioflavin T Binding Assay and Transmission Electron Microscopy Examination

The inhibiting potential of the curcumin and coumarin-chalcone derivatives on A*β* amyloid aggregation was assessed using A*β*_42_ peptide (AnaSpec, Fremont, CA, USA). In brief, the tested compound (5–20 *μ*M) was added to a buffer solution (150 mM NaCl, 20 mM Tris-HCl (pH 8.0)) containing A*β*_42_ peptide (5 *μ*M) and incubated at 37°C for 48 h. Thioflavin T (10 *μ*M) (Sigma-Aldrich) then was added, and the mixture was incubated for 5 min at room temperature. Subsequently, the fluorescence intensity was quantified at 420/485 nm of excitation/emission wavelengths using a FLx800 microplate reader (Bio-Tek, Winooski, VT, USA). EC_50_ was computed as described.

The potential of Congo red and coumarin-chalcone derivatives in inhibiting tau misfolding was assessed using the *E. coli*-derived *Δ*K280 tau_RD_ protein [21]. In brief, *Δ*K280 tau_RD_ protein (20 *μ*M) was incubated with the tested compound (1−10 *μ*M) in buffer (150 mM NaCl, 20 mM Tris-HCl (pH 8.0)) at 37°C for 48 h to form aggregates. Thioflavin T (5 *μ*M) was then added, and the mixture was incubated at room temperature for 25 min. The fluorescence intensity of the samples was measured, and the EC_50_ was computed as described.

To investigate A*β* and *Δ*K280 tau_RD_ aggregates, samples of the fibrils formed with and without treatment of a tested compound were placed on a 200-mesh copper (holey-carbon) grid and visualized using a JEM-1230 transmission electron microscope (TEM) (JEOL, Tokyo, Japan) at an accelerating voltage of 100 kV.

### 2.5. Cell Culture

Human 293-derived Flp-In-293 cells (Invitrogen, Madison, WI, USA) were cultivated in Dulbecco's modified Eagle's medium (DMEM) containing 10% foetal bovine serum (FBS) (Thermo Fisher Scientific). Human neuroblastoma SH-SY5Y-derived A*β*-GFP [23] and ∆K280 tau_RD_-DsRed cells [24] were kept in DMEM/nutrient mixture F12 (DMEM/F12) containing 10% FBS, 5 *μ*g/ml blasticidin, and 100 *μ*g/ml hygromycin (InvivoGen, San Diego, CA, USA). Doxycycline (Sigma-Aldrich) was applied to induce the expression of A*β*-GFP (5 *μ*g/ml doxycycline) or ∆K280 tau_RD_-DsRed (2 *μ*g/ml doxycycline).

### 2.6. CRE-GFP Reporter Construct and 293 Reporter Cells

Using the pCRE-MetLuc2-Reporter plasmid (Clontech, Mountain View, CA, USA) as a template, the DNA fragment containing the cAMP response element (CRE) motifs and TATA-like promoter (*P*_TAL_) flanked by *Mlu*I and *Age*I restriction sites was amplified by PCR using sense (ACGCGTGCACCAGACAGTGACGTC, *Mlu*I site underlined) and antisense (ACCGGTCTGCTTCATCCCCGTGG, *Age*I site underlined) primers. The amplified CRE-*P*_TAL_ promoter fragment was cloned into pGEM-T Easy (Promega, Madison, WI, USA) and sequenced. The CRE-*P*_TAL_-containing *Mlu*I/*Age*I fragment and GFP-containing *Age*I/*Not*I fragment (from pEGFP-N1; Clontech) were subcloned into *Mlu*I- and *Not*I-digested pcDNA5/FRT/TO (Novagen, Madison, WI, USA). The resulting construct with CRE-*P*_TAL_-driving GFP was used to establish 293 GFP reporter cells by targeting insertion into Flp-In-293 cells according to the supplier's instructions (Invitrogen). The cells were grown in a medium containing 5 *μ*g/ml blasticidin and 100 *μ*g/ml hygromycin.

### 2.7. CRE-GFP 293 Reporter Cell Assay

On day 1, CRE-GFP 293 cells were seeded into 48-well plates (5 × 10^4^/well). After 24 h, cells were treated with Ca^2+^ ionophore (2 *μ*M) (Sigma-Aldrich) for 0–24 h. Cell viability was measured using 3,(4,5-dimethylthiazol-2-yl)-2,5-diphenyltetrazolium bromide (MTT) assay (Sigma-Aldrich). The absorbance of the insoluble purple formazan dye was quantified at 570 nm on a *μ*Quant microplate spectrophotometer (Bio-Tek). In addition, the cells were treated with the Ca^2+^ ionophore A23187 (0.5–10 *μ*M) (Sigma-Aldrich) for 5 h, followed by analysis of GFP fluorescence by flow cytometry (Becton–Dickinson, Franklin Lakes, NJ, USA) with excitation/emission wavelengths at 488/507 nm. To monitor CREB activation, cells were treated with forskolin or coumarin-chalcone derivatives (5–10 *μ*M) in the presence or absence of Ca^2+^ ionophore (2 *μ*M) for 5 h, and GFP fluorescence was analysed.

### 2.8. High Content Analysis of A*β*-GFP, ∆K280 tau_RD_-DsRed, and ROS Fluorescence

On day 1, A*β*-GFP or ∆K280 tau_RD_-DsRed SH-SY5Y cells were seeded into 96-well plates (2.5 × 10^4^/well) and treated with retinoic acid (10 *μ*M; Sigma-Aldrich) to prompt neuronal differentiation [28]. On day 2, A*β*-GFP cells were pretreated with curcumin or coumarin-chalcone derivatives (1.2–5 *μ*M) for 8 h, followed by doxycycline (5 *μ*g/ml) treatment to induce A*β*-GFP expression. ∆K280 tau_RD_-DsRed cells were treated with Congo red or coumarin-chalcone derivatives (2.5–10 *μ*M) and doxycycline (2 *μ*g/ml). On day 8, cells were stained with Hoechst 33342 (0.1 *μ*g/ml; Sigma-Aldrich) for 30 min, and cell images were automatically captured at excitation/emission wavelengths of 482/436 nm (GFP fluorescence) or 543/593 nm (DsRed fluorescence) using a high content analysis (HCA) system (Micro Confocal High-Content Imaging System). Image Acquisition and Analysis Software (MetaXpress) (Molecular Devices, Sunnyvale, CA, USA) was used to analyse the images. CellROX Orange (5 *μ*M; Molecular Probes, Eugene, OR, USA) (for A*β*-GFP cells) or dichloro-dihydro-fluorescein diacetate (DCFH-DA, 10 *μ*M; Invitrogen) (for ∆K280 tau_RD_-DsRed cells) was added to the cells and incubated at 37°C for 30 min. The HCA system was used to measure intracellular ROS at 531/593 nm (CellROX Orange fluorescence) or 482/536 nm (DCFH-DA fluorescence) of excitation/emission wavelengths.

### 2.9. High Content Analysis of Neurite Outgrowth

On day 1, A*β*-GFP or ∆K280 tau_RD_-DsRed SH-SY5Y cells were plated into 24-well plates (6 × 10^4^/well) followed by addition of retinoic acid. On day 2, cells were treated with coumarin-chalcone derivatives (5 or 10 *μ*M) and with doxycycline (5 or 2 *μ*g/ml) to induce the expression of A*β*-GFP or ∆K280 tau_RD_-DsRed. On day 8, 4% paraformaldehyde, 0.1% Triton X-100, and 3% BSA were used to fix, permeabilize, and block cells, respectively. Subsequently, the cells were stained with TUBB3 (neuronal class III *β*-tubulin) primary antibody (1 : 1000; Covance, Princeton, NJ, USA) at 4°C overnight. Goat anti-rabbit Alexa Fluor 555 secondary antibody (1 : 1000; Thermo Fisher Scientific) was then added, followed by a 3 h incubation at room temperature. Following staining nuclei with 4′,-6-diamidino-2-phenylindole (DAPI) (0.1 *μ*g/ml; Sigma-Aldrich), the neurons were imaged by using the HCA system and analysed by using the Neurite Outgrowth Application Module (MetaXpress; Molecular Devices).

### 2.10. Caspase 1, Acetylcholinesterase, and Real-Time PCR Assays

A*β*-GFP or ∆K280 tau_RD_-DsRed SH-SY5Y cells were seeded into 6-well plates (5 × 10^5^/well) and treated with retinoic acid, coumarin-chalcone derivatives, and doxycycline as previously described. On day 8, cells were collected and lysates were prepared with six freeze/thaw cycles. The supernatant was collected by centrifugation, followed by measurement of the caspase 1 activity in 50 *μ*g aliquots of cell extract using the ICE fluorometric assay kit (BioVision, Milpitas, CA, USA). Following incubation of the mixture at 37°C for 2 h, fluorescence was measured with excitation/emission wavelengths at 400/505 nm (FLx800 fluorescence microplate reader). In addition, the collected cells were lysed by sonication. Acetylcholinesterase (AChE) activity in the supernatant was determined in 10 *μ*g aliquots of cell extracts using the AChE activity assay kit (Sigma-Aldrich) according to the manufacture's protocol. Finally, the absorbance at 412 nm was measured using a Multiskan GO spectrophotometer (Thermo Fisher Scientific).

To measure the A*β*-GFP/tau_RD_-DsRed RNA expression on day 8, total RNA was extracted from collected cells and reverse-transcribed to cDNA (SuperScript III reverse transcriptase; Invitrogen, Waltham, MA, USA). Real-time quantitative PCR was performed using 100 ng of cDNA and the gene-specific TaqMan fluorogenic probe PN4331348 (EGFP) or customized Assays-by-Design probe for DsRed [24] and 4326321E (HPRT1) (StepOnePlus Real-time PCR system, Applied Biosystems, Foster City, CA, USA). The RNA expression level was calculated using the formula 2^*Δ*Ct^, ΔC_T_ = C_T_ (control) − C_T_ (target), in which C_T_ indicates the cycle threshold.

### 2.11. Kinase Inhibitor Treatment

To monitor the cAMP-mediated signal transduction pathway, CRE-GFP 293 cells in 6-well plates (1 × 10^6^/well) were pretreated with kinase inhibitor H-89 (LC Laboratories, Woburn, MA, USA), KN-62 (Cayman Chemical, Ann Arbor, MI, USA), U0126 (LC Laboratories), or wortmannin (LC Laboratories) (10 *μ*M) for 4 h before forskolin or LM-021 (10 *μ*M) addition for 5 h. GFP fluorescence/protein and total/phosphorylated PKA, CaMKII, ERK, PI3K, and CREB levels were examined. In addition, A*β*-GFP or ∆K280 tau_RD_-DsRed SH-SY5Y cells were treated with retinoic acid (10 *μ*M) on day 1, followed by the addition of LM-021 (5 or 10 *μ*M) and doxycycline (5 or 2 *μ*g/ml) on day 2, as described. Kinase inhibitors (10 *μ*M) were added on day 6. On day 8, the cells were collected for protein expression analysis of BDNF, BCL2 (BCL2 apoptosis regulator), and BAX (BCL2 associated X, apoptosis regulator), and total/phosphorylated PKA, CaMKII, ERK, PI3K, and CREB. Also, cells were stained with DAPI and assessed for neurite outgrowth as described above.

### 2.12. Western Blot Analysis

Total proteins from CRE-GFP 293 and A*β*-GFP, ∆K280 tau_RD_-DsRed SH-SY5Y cells were extracted using lysis buffer (50 mM Tris-HCl (pH 8.0), 150 mM NaCl, 1 mM EDTA (pH 8.0), 1 mM EGTA (pH 8.0), 0.1% SDS, 0.5% sodium deoxycholate, 1% Triton X-100) containing phosphatase and protease inhibitor cocktails (Sigma-Aldrich). Protein concentrations were measured using a protein assay kit (Bio-Rad, Hercules, CA, USA), and 20 *μ*g of proteins was separated with 10% SDS-PAGE and blotted onto polyvinylidene difluoride (PVDF) membranes (Sigma-Aldrich). After blocking, the membrane blot was incubated with primary antibody against GFP (1 : 500; Santa Cruz Biotechnology, Santa Cruz, CA, USA), PKA (1 : 1000; R&D Systems, Minneapolis, MN, USA), p-PKA (T197) (1 : 1000; Cell Signaling, Danvers, MA, USA), ERK (1 : 500; Cell Signaling), p-ERK (T202/Y204) (1 : 500; Cell Signaling), CaMKII (1 : 1000; Santa Cruz Biotechnology), p-CaMKII (T286) (1 : 1000; Cell Signaling), CREB (1 : 1000; Santa Cruz Biotechnology), p-CREB (S133) (1 : 1000; Millipore, Billerica, MA, USA), BDNF (1 : 500; Santa Cruz Biotechnology), BCL2 (1 : 500; BioVision), BAX (1 : 500; BioVision), GAPDH (glyceraldehyde-3-phosphate dehydrogenase, as a loading control) (1 : 1000; MDBio, Taipei, Taiwan), or *β*-tubulin (1 : 1000, Sigma-Aldrich, as a loading control). The immune complexes were visualized using horseradish peroxidase-conjugated goat anti-mouse or goat anti-rabbit IgG antibody (1 : 5000, GeneTex, Irvine, CA, USA) and a chemiluminescent substrate (Millipore).

### 2.13. Mouse Pharmacokinetics Study and Brain/Plasma Ratio Determination

Male Crl:CD-1 (ICR) mice (8 weeks old) weighing 25–30 g were housed in cages with *ad libitum* access to food and drinking water on a 12 : 12 h light-dark cycle. All animal experiments were performed in accordance with the guidelines approved by the Rosetta Pharmamate Institutional Animal Care and Use Committee (IACUC Approval No: AF20015). Mice were randomly divided into 7 subgroups (6 time points and 1 vehicle control; *n* = 3 per subgroup). LM-021 (in 5% DMSO and 19% hydroxypropyl-*β*-cyclodextrin) was administered intravenously (IV) at 5 mg/kg dose in volume of 10 ml/kg. Mice were euthanized in a CO_2_ chamber at 0.25, 0.5, 1, 2, 4, or 8 h postdose. Whole blood was collected via facial veins in heparin-coated polypropylene tubes, and plasma was obtained by centrifugation (1000 × g for 15 min at 4°C) within 60 min of collection. After cerebral vasculature perfusion through the left ventricle with normal saline for 5 min, the whole brain was quickly excised and weighed. Plasma and brain samples were rapidly frozen and stored at −70°C until analysis (see supplementary materials).

Pharmacokinetic (PK) parameters, including plasma concentration following IV dose (C_0_), elimination half-life (T_1/2_), mean residence time (MRT), and area under the concentration- (AUC-) time curve from time 0 extrapolated to infinity (AUC_(0-∞)_), were estimated using noncompartmental pharmacokinetic parameter analysis with Phoenix WinNonlin software (version 8.2, Pharsight Corporation, Mountain View, CA, USA). In addition, the ratio of brain/plasma was calculated from the area under the curve for brain and plasma concentrations.

### 2.14. Statistical Analysis

Data are expressed as the mean ± standard deviation of the results from three independent experiments. Comparisons between groups were analysed using a two-tailed Student's *t*-test or one-way ANOVA (analysis of variance) with a post hoc Tukey test where appropriate. A *p* < 0.05 was considered to be statistically significant.

## 3. Results

### 3.1. Tested Coumarin-Chalcone Compounds and Biochemical A*β*/Tau Aggregation Inhibition

**Figure 1:**
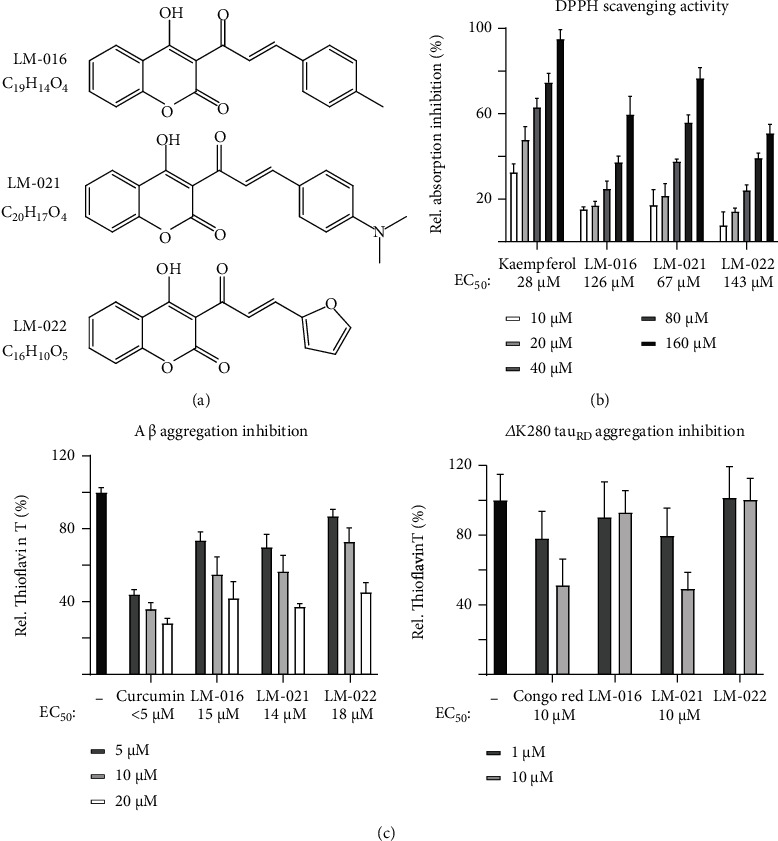
Coumarin-chalcone derivatives. (a) Structure, formula, and molecular weight of tested LM-016, LM-021, and LM-022. (b) Radical-scavenging activity of kaempferol (as a positive control), LM-016, LM-021, and LM-022 (10−160 *μ*M) evaluated by using DPPH (*n* = 3). The EC_50_ values are shown below. (c) A*β* aggregation-inhibitory effects of curcumin (as a positive control), LM-016, LM-021, and LM-022 (5–20 *μ*M) and *Δ*K280 tau_RD_ aggregation-inhibitory effects of Congo red (as a positive control), LM-016, LM-021, and LM-022 (1–10 *μ*M) evaluated by the Thioflavin T assay (*n* = 3). To normalize, the Thioflavin T fluorescence of A*β*_42_/*Δ*K280 tau_RD_ without compound treatment was set as 100%. The EC_50_ values are shown in the following.

Coumarin-chalcone derivatives have been shown to upregulate the CREB pathway in AD and polyQ cell models [20–22]. Here, we examined three synthetic coumarin-chalcone derivatives LM-016, LM-021, and LM-022 (Figure 1(a)), all of which fulfilled Lipinski's criteria for oral bioavailability on the basis of molecular weight, hydrogen bond donors, hydrogen bond acceptors, and calculated octanol/water partition coefficient [29] (Figure S1A). Given that all compounds have a polar surface area smaller than 90 Å^2^, they were predicted to diffuse across the BBB [30], which was also recommended by the online BBB predictor [31] (Figure S1A).

Inhibition of amyloid aggregation and oxidative stress are considered important treatment approaches for AD. The free radical-scavenging activity of these coumarin-chalcone derivatives was examined using DPPH as a substrate. A natural antioxidant kaempferol [32] was used as a positive control. EC_50_ values of kaempferol, LM-016, LM-021, and LM-022 were 28, 126, 181, and 143 *μ*M, respectively (Figure 1(b)). The A*β* or ∆K280 tau_RD_ aggregation-inhibitory effect of the test compounds was measured using Thioflavin T, a dye widely used to examine misfolded protein aggregates [33]. Curcumin or Congo red, known to reduce amyloid aggregation [34, 35], was included as a positive control. As shown in Figure 1(c), curcumin (EC_50_ < 5 *μ*M), LM-016 (EC_50_ = 15 *μ*M), LM-021(EC_50_ = 14 *μ*M), and LM-022 (EC_50_ = 18 *μ*M) treatment significantly inhibited A*β* aggregation in the Thioflavin T fluorescence assay. In addition, Congo red and LM-021 reduced *Δ*K280 tau_RD_ aggregation, with EC_50_ of 10 *μ*M (Figure 1(c)). TEM examination of A*β* and tau aggregate structures also displayed reduced amyloid aggregates with LM-021 treatment (10 *μ*M) (Figure S1B). The results showed that LM-021 directly hampered both A*β* and *Δ*K280 tau_RD_ aggregate formation.

### 3.2. Coumarin-Chalcone Derivatives Increase CRE-Mediated Gene Expression

**Figure 2:**
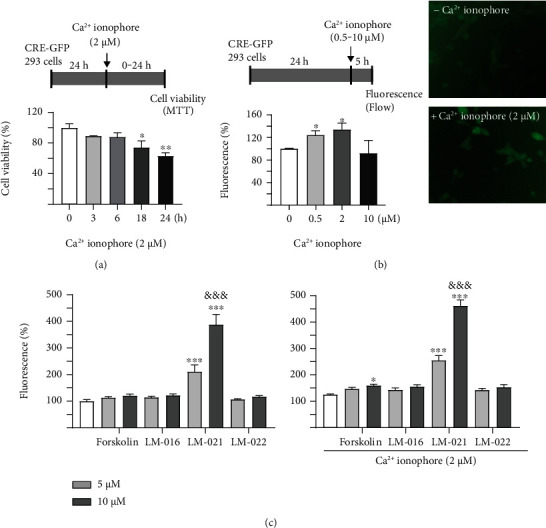
CRE fluorescence reporter assay. (a) Experimental flow chart to determine optimal Ca^2+^ ionophore treatment time. CRE-GFP 293 cells were treated with Ca^2+^ ionophore (2 *μ*M) for 0–24 h. Cell viability was assessed by MTT assay (*n* = 3; two-tailed Student's *t*-test; ^∗^*p* < 0.05 and ^∗∗^*p* < 0.01). (b) Left: experimental flow chart to determine optimal Ca^2+^ ionophore treatment concentration. CRE-GFP 293 cells were treated with Ca^2+^ ionophore (0.5–10 *μ*M) for 5 h. GFP fluorescence was assessed by flow cytometry (*n* = 3; two-tailed Student's *t*-test; ^∗^*p* < 0.05). Right: Flp-In CRE fluorescence reporter cells with or without Ca^2+^ ionophore (2 *μ*M) treatment for 5 h. (c) Fluorescence analysis of CRE reporter cells untreated or treated with Ca^2+^ ionophore (2 *μ*M) and forskolin, LM-016, LM-021, or LM-022 (5–10 *μ*M) for 5 h. GFP fluorescence was assessed by flow cytometry (*n* = 3; one-way ANOVA with a post hoc Tukey test). *p* values: compound treated vs. untreated cells (^∗^*p* < 0.05 and ^∗∗∗^*p* < 0.001), or 10 *μ*M compound-treated vs. 5 *μ*M compound-treated cells (^&&&^*p* < 0.001).

To monitor the activation of CREB, a plasmid with GFP reporter driven by the CRE motif-TATA-like promoter (Figure S2A) was used to establish Flp-In 293 GFP reporter cells. As Ca^2+^ influx triggers phosphorylation of CREB [36], the reporter cell was tested with 2 *μ*M Ca^2+^ ionophore for 3–24 h and 0.5–10 *μ*M Ca^2+^ ionophore for 5 h to obtain the optimized experimental condition. No significant cell death was observed after treatment with 2 *μ*M Ca^2+^ ionophore for 3–6 h (Figure 2(a)). Thus, Ca^2+^ ionophore treatment for 5 h was selected for the following test. As shown in Figure 2(b), treatment with 0.5–2 *μ*M Ca^2+^ ionophore for 5 h stimulated CRE-motif-driven GFP expression. CRE-GFP reporter cells treated with 2 *μ*M Ca^2+^ ionophore for 5 h were then used to test the studied coumarin-chalcone derivatives (Figure S2B). As shown in Figure 2(c), LM-021 (5–10 *μ*M) significantly activated CRE-motif-driven GFP expression in the presence or absence of Ca^2+^ ionophore in a dose-dependent manner. Forskolin (10 *μ*M), an activator of adenylyl cyclase [37], significantly increased CRE-motif-driven GFP expression in the presence of Ca^2+^ ionophore.

### 3.3. Coumarin-Chalcone Derivatives Inhibit A*β* Aggregation and Promote Neurite Outgrowth

**Figure 3:**
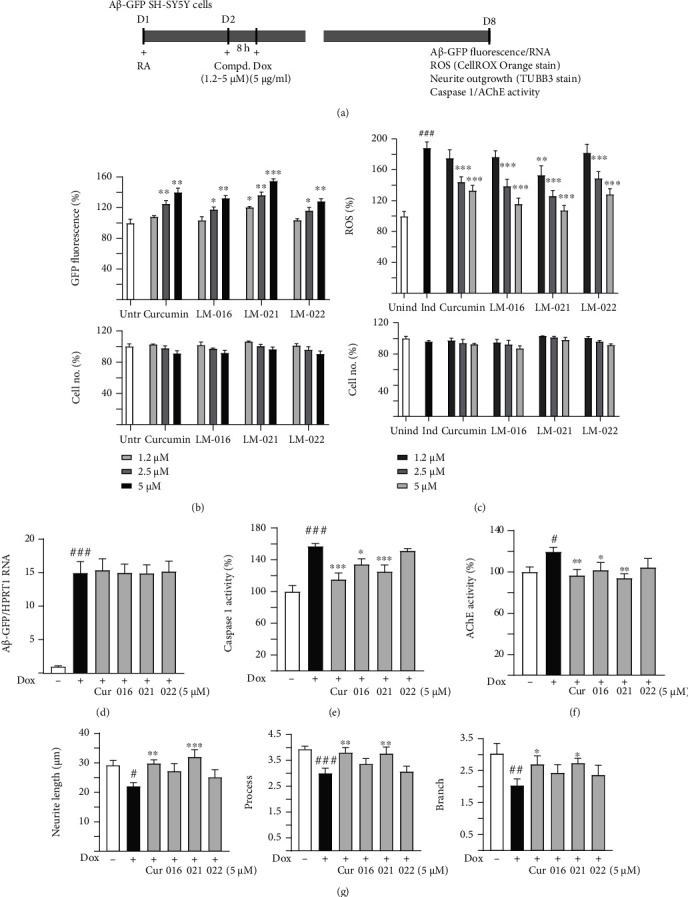
Neuroprotective effects of coumarin-chalcone derivatives on A*β*-GFP-expressing SH-SY5Y cells. (a) Experimental flow chart. On day 1, cells were plated with retinoic acid (RA, 10 *μ*M) being added to the culture medium. On day 2, the cells were treated with curcumin, LM-016, LM-021, or LM-022 (1.2–5 *μ*M) for 8 h, followed by adding doxycycline (Dox, 5 *μ*g/ml) to induce A*β*-GFP expression for 6 days. On day 8, A*β*-GFP fluorescence, ROS (CellROX Orange staining), neurite outgrowth (TUBB3 staining), and caspase 1/AChE activities were measured. (b) Analysis of GFP fluorescence with curcumin, LM-016, LM-021, or LM-022 (1.2–5 *μ*M) treatment (*n* = 3; two-tailed Student's *t*-test; ^∗^*p* < 0.05, ^∗∗^*p* < 0.01, and ^∗∗∗^*p* < 0.001). The cell numbers counted for each treatment are shown in the following. For normalization, the GFP fluorescence/cell number of untreated cells (Untr) was set as 100%. (c) ROS assay with curcumin, LM-016, LM-021, or LM-022 (1.2–5 *μ*M) treatment (*n* = 3). The cell numbers counted for each treatment are shown in the following. To normalize, the ROS/cell number of uninduced cells (Dox-) was set as 100%. (d) A*β*-GFP RNA, (e) caspase 1, and (f) AChE activity assays with curcumin, LM-016, LM-021, or LM-022 (5 *μ*M) treatment (*n* = 3). To normalize, the caspase 1/AChE activities of uninduced cells (Dox-) were set as 100%. (g) Neurite outgrowth (length, process, and branch) assay with curcumin, LM-016, LM-021, or LM-022 (5 *μ*M) treatment (*n* = 3). (c–g) *p* values: induced (Dox+) vs. uninduced (Dox-) cells (^#^*p* < 0.05, ^##^*p* < 0.01, and ^###^*p* < 0.001), or compound-treated vs. untreated (Dox+) cells (^∗^*p* < 0.05, ^∗∗^*p* < 0.01, and ^∗∗∗^*p* < 0.001) (one-way ANOVA with a post hoc Tukey test).

Tet-On A*β*-GFP SH-SY5Y cells [23], a reporter cell reflecting the A*β* misfolding level through GFP fluorescence intensity, were used to determine the ability of coumarin-chalcone derivatives to inhibit A*β* aggregation (Figure 3(a)). Curcumin was used as a positive control. The green fluorescence intensity of 2.5–5 *μ*M curcumin-pretreated cells was significantly higher than that of untreated cells. Treatment with LM-016 at 2.5–5 *μ*M, LM-021 at 1.2–5 *μ*M, or LM-022 at 2.5–5 *μ*M also significantly augmented the green fluorescence intensity (Figure 3(b)). Curcumin, LM-016, LM-021, and LM-022 had an EC_50_ value of 6.1, 7.0, 5.1, and 8.2 *μ*M, respectively, in A*β* aggregation inhibition (Figure S3A). In oxidative stress analysis, A*β*-GFP expression induced significantly elevated ROS levels in A*β*-GFP-expressing SH-SY5Y cells, while treatment with curcumin at 2.5–5 *μ*M, LM-016 at 2.5–5 *μ*M, LM-021 at 1.2–5 *μ*M, or LM-022 at 2.5–5 *μ*M effectively decreased the ROS levels caused by A*β* overexpression (Figures 3(c) and S3B). These results suggest that these three coumarin-chalcone derivatives not only impeded A*β* aggregation but also reduced ROS overproduced by A*β*. Treatment with curcumin, LM-016, LM-021, or LM-022 did not significantly modify A*β*-GFP RNA levels (Figure 3(d)), suggesting that the increases in fluorescence intensity and ROS were not caused by changes in gene expression.

A*β* aggregation increases AChE activity to accelerate A*β* fibril formation [38] and reduces neurite outgrowth [39]. Increased caspase 1 is linked with axonal degeneration in AD patients [40]. Therefore, we evaluated the neuroprotective effects of LM-016, LM-021, and LM-022 by assessing AChE/caspase 1 activities and neurite outgrowth. The caspase 1 and AChE activities were significantly increased by A*β* overexpression and reduced by treatment with curcumin, LM-016, or LM-021 (5 *μ*M) compared to untreated cells (Figures 3(e) and 3(f)). In addition, the A*β* overexpression significantly reduced neurite length, processes, and branches. Treatment with curcumin or LM-021 (5 *μ*M) successfully ameliorated the impaired neurite length, processes, and branches (Figures 3(g) and S3C).

### 3.4. Coumarin-Chalcone Derivatives Inhibit Tau Aggregation and Promote Neurite Outgrowth

**Figure 4:**
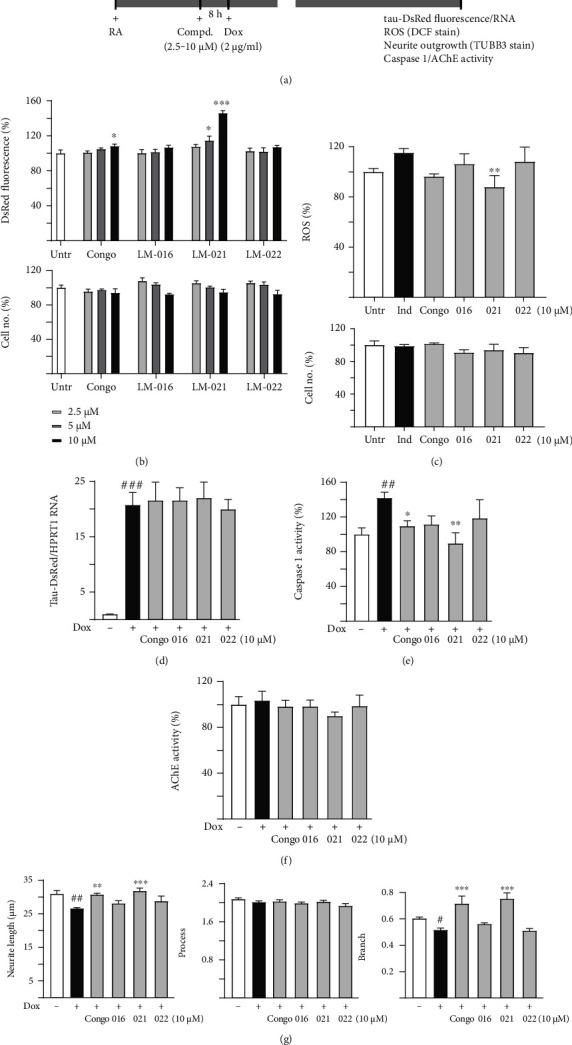
Neuroprotective effects of coumarin-chalcone derivatives on *Δ*K280 tau_RD_-DsRed-expressing SH-SY5Y cells. (a) Experimental flow chart. On day 1, cells were plated, with retinoic acid (RA, 10 *μ*M) being added to the culture medium. On day 2, Congo red, LM-016, LM-021, or LM-022 (2.5–10 *μ*M) were added to the cells for 8 h, followed by treatment with doxycycline (Dox, 2 *μ*g/ml) to induce *Δ*K280 tau_RD_-DsRed expression for 6 days. On day 8, *Δ*K280 tau_RD_-DsRed fluorescence, ROS (DCF staining), neurite outgrowth (TUBB3 staining), and caspase 1/AChE activities were measured. (b) Analysis of DsRed fluorescence with Congo red, LM-016, LM-021, or LM-022 (2.5–10 *μ*M) treatment (*n* = 3; two-tailed Student's *t*-test; ^∗^*p* < 0.05 and ^∗∗∗^*p* < 0.001). The cell numbers counted in each treatment are displayed in the following. The DsRed fluorescence/cell number of untreated cells (Untr) was set as 100% for normalization. (c) ROS assay with Congo red, LM-016, LM-021, or LM-022 (10 *μ*M) treatment (*n* = 3). The cell numbers counted in each treatment are displayed in the following. The relative ROS/cell number of uninduced cells (Dox-) was normalized (100%). (d) Tau_RD_-DsRed RNA, (e) caspase 1, and (f) AChE activity assays with Congo red, LM-016, LM-021, or LM-022 (10 *μ*M) treatment (*n* = 3). For normalization, the caspase 1/AChE activities of uninduced cells (Dox-) were set as 100%. (g) Neurite outgrowth (length, process, and branch) assay with Congo red, LM-016, LM-021, or LM-022 (10 *μ*M) treatment (*n* = 3). (c–g) *p* values: induced (Dox+) vs. uninduced (Dox-) cells (^#^*p* < 0.05 and ^##^*p* < 0.01), or compound-treated vs. untreated (Dox+) cells (^∗^*p* < 0.05, ^∗∗^*p* < 0.01, and ^∗∗∗^*p* < 0.001) (one-way ANOVA with a post hoc Tukey test).

The ability of coumarin-chalcone derivatives to inhibit tau aggregation and promote neurite outgrowth was tested by using Tet-On ∆K280 tau_RD_-DsRed SH-SY5Y cells [24] (Figure 4(a)). The poorly folded *Δ*K280 tau_RD_ caused misfolding of fused DsRed, leading to decreased DsRed fluorescence [41]. For comparison, Congo red was included as a positive control. Treatment with Congo red at 10 *μ*M and LM-021 at 5–10 *μ*M significantly elevated the DsRed fluorescence intensity (Figure 4(b)). Based on the measured concentrations, Congo red, LM-016, LM-021, and LM-022 had extrapolated EC_50_ values of 57, 70, 10, and 73 *μ*M, respectively, in inhibiting ∆K280 tau_RD_ aggregation (Figure S4A). ∆K280 tau_RD_-DsRed expression elevated ROS levels, although the difference did not reach statistical significance. LM-021 at 10 *μ*M effectively reduced the ROS level elevated by ∆K280 tau_RD_ overexpression (Figures 4(c) and S4B). Treatment with Congo red or LM-021 did not significantly alter the ∆K280 tau_RD_-DsRed RNA level (Figure 4(d)).

The neuroprotective effects of Congo red and coumarin-chalcone derivatives on caspase 1/AChE activities and neurite outgrowth were also examined. As shown in Figure 4(e), the overexpression of ∆K280 tau_RD_ significantly enhanced caspase 1 activity, and treatment with Congo red or LM-021 (10 *μ*M) lowered caspase 1 activity. In contrast, AChE activity was not significantly changed by ∆K280 tau_RD_ overexpression or compound treatment (Figure 4(f)). ∆K280 tau_RD_ overexpression significantly decreased neurite length and branches. Treatment with Congo red or LM-021 0(10 *μ*M) successfully ameliorated the deficits in neurite length and branches (Figures 4(g) and S4C).

### 3.5. Regulatory Targets of CREB Phosphorylation by LM-021 in CRE-GFP Reporter Cells

**Figure 5:**
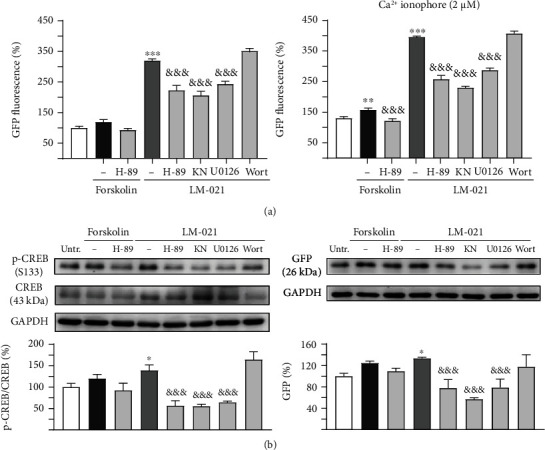
LM-021-mediated kinase activation in CRE-GFP reporter cells. (a) Fluorescence analysis of CRE reporter cells without and with kinase inhibitor, Ca^2+^ ionophore, and/or forskolin or LM-021 treatment. GFP fluorescence was assessed by flow cytometry (*n* = 3). To normalize, the GFP fluorescence level in untreated cells was set as 100%. (b) p-CREB and GFP levels analysed by immunoblot using GAPDH as a loading control (*n* = 3). To normalize, the protein expression level in untreated cells was set as 100%. *p* values: compound treated vs. untreated cells (^∗^*p* < 0.05, ^∗∗^*p* < 0.01, and ^∗∗∗^*p* < 0.001), or kinase inhibitor-treated vs. untreated cells (^&&&^: *p* < 0.001) (one-way ANOVA with a post hoc Tukey test).

To further investigate if LM-021 employs its effect by increasing the phosphorylation of CREB, the inhibitor H-89 (PKA inhibitor), KN-62 (CaMKII inhibitor), U0126 (ERK inhibitor), or wortmannin (PI3K inhibitor) (10 *μ*M) was added to CRE-GFP reporter cells 4 h before addition of coumarin-chalcone derivatives (10 *μ*M) and/or Ca^2+^ ionophore (2 *μ*M) (Figure S5). As shown in Figure 5(a), forskolin (positive control) significantly activated CRE-motif-driven GFP expression in the presence of Ca^2+^ ionophore over that of untreated cells, while H-89 treatment attenuated the GFP level. In addition, LM-021 significantly activated CRE-mediated transcription in the presence or absence of Ca^2+^ ionophore. The kinase inhibitors H-89, KN-62, and U0126 but not wortmannin attenuated the GFP levels in the presence or absence of Ca^2+^ ionophore.

The p-CREB (S133) and GFP protein levels in CRE-GFP reporter cells without Ca^2+^ ionophore addition were further examined by immunoblotting using specific antibodies. As shown in Figure 5(b), the increased p-CREB and GFP levels were also significantly reduced in CRE-GFP reporter cells after treatment with H-89, KN-62, or U0126 but not wortmannin. The results demonstrate that LM-021 stimulated cAMP-mediated transcription through the PKA, CaMKII, and ERK pathways.

### 3.6. Regulation of CREB Phosphorylation by LM-021 in A*β*-GFP SH-SY5Y Cells

**Figure 6:**
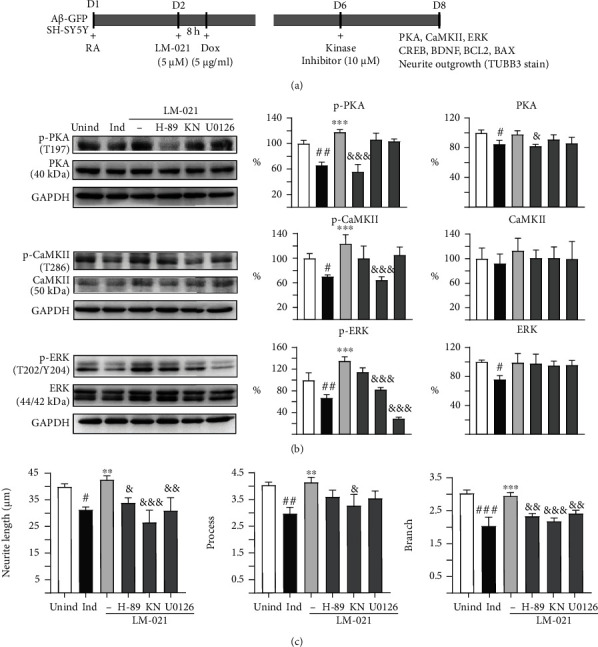
LM-021-mediated kinase activation in A*β*-GFP-expressing SH-SY5Y cells. (a) Experimental flow chart. On day 1, cells were plated with retinoic acid (RA, 10 *μ*M) being added to the culture medium. On day 2, LM-021 (5 *μ*M) was added to the cells for 8 h, followed by treatment of doxycycline (Dox, 5 *μ*g/ml) to induce A*β*-GFP expression. Kinase inhibitors (10 *μ*M) were added to the cells on day 6. On day 8, p-PKA, p-CaMKII, p-ERK, p-CREB, BDNF, BCL, and BAX levels, as well as neurite outgrowth (TUBB3 staining), were measured. (b) p-PKA, p-CaMKII, and p-ERK protein levels determined by immunoblot using GAPDH as a loading control (*n* = 3). To normalize, the protein expression level in untreated cells was set as 100%. (c) Measurements of neurite outgrowth (length, process, and branch) (*n* = 3). *p* values: induced vs. uninduced cells (^#^*p* < 0.05, ^##^*p* < 0.01, and ^###^*p* < 0.001), LM-021-treated vs. untreated cells (^∗^*p* < 0.05, ^∗∗^*p* < 0.01, and ^∗∗∗^*p* < 0.001), or kinase inhibitor-treated vs. untreated cells (^&^*p* < 0.05, ^&&^*p* < 0.01, and ^&&&^*p* < 0.001) (one-way ANOVA with a post hoc Tukey test).

CREB plays an important role in cell survival and synaptic activity by upregulating BCL2 [42] and BDNF [36], following phosphorylation at Ser133. CREB-regulated BDNF and BCL2 pathways are compromised in the hippocampus of *APP* transgenic mice, and the overexpression of CREB protects rat primary hippocampal neurons against A*β*-induced apoptosis [15]. To further investigate the CREB-mediated neuroprotective potential of LM-021, we applied the kinase inhibitor H-89, KN-62, or U0126 to LM-021-treated A*β*-GFP-expressing SH-SY5Y cells (Figure 6(a)). Protein expression levels of PKA, CaMKII, ERK, CREB, BDNF, BCL2, and BAX were examined. As shown in Figure 6(b), while the level of CaMKII was not notably affected, overexpression of A*β*-GFP downregulated PKA and ERK. LM-021 treatment rescued the reduction in PKA and ERK, although not significantly. The overexpression of A*β*-GFP reduced p-PKA, p-CaMKII, and p-ERK protein levels, and treatment with LM-021 significantly upregulated p-PKA, p-CaMKII, and p-ERK, whereas H-89 treatment mitigated the increase of p-PKA, KN-62 treatment mitigated the upregulation of p-CaMKII, and KN-62 or U0126 treatment normalized p-ERK.

Moreover, induced expression of A*β*-GFP reduced p-CREB, CREB, pro-BDNF, m-BDNF, and BCL2 and increased BAX protein levels, and treatment with LM-021 significantly increased p-CREB, CREB, pro-BDNF, m-BDNF, and BCL2 and reduced BAX protein levels. Treatment with H-89, KN-62, or U0126 attenuated the increase in p-CREB, pro-BDNF, m-BDNF, and BCL2 and reduced the decrease in BAX (Figure S6A). In A*β*-GFP-expressing SH-SY5Y cells, LM-021 salvaged the impaired neurite length, processes, and branches, whereas H-89, KN-62, or U0126 treatment counteracted these improvements (Figures 6(c) and S6B).

### 3.7. Regulation of CREB Phosphorylation by LM-021 in ∆K280 Tau_RD_-DsRed SH-SY5Y Cells

**Figure 7:**
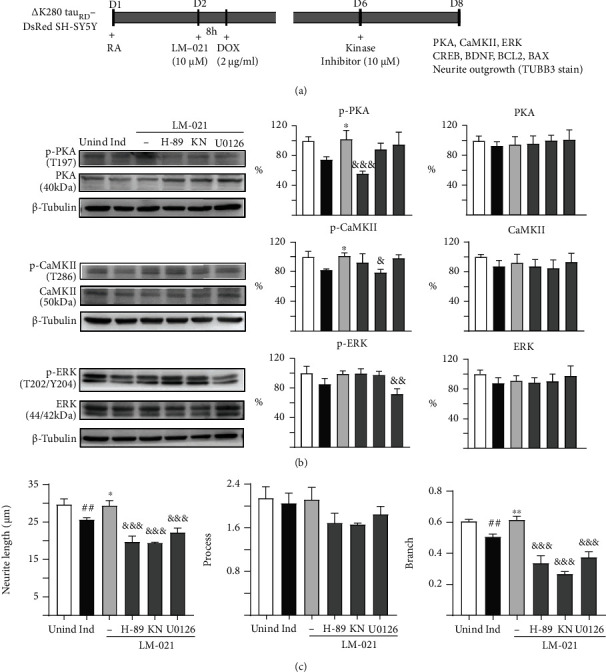
LM-021-mediated kinase activation in *Δ*K280 tau_RD_-DsRed-expressing SH-SY5Y cells. (a) Experimental flow chart. On day 1, cells were plated with retinoic acid (RA, 10 *μ*M) being added to the culture medium. On day 2, LM-021 (10 *μ*M) was added to the cells for 8 h, followed by adding doxycycline (Dox, 2 *μ*g/ml) to induce *Δ*K280 tau_RD_-DsRed expression. Kinase inhibitors (10 *μ*M) were added to the cells on day 6. On day 8, p-PKA, p-CaMKII, p-ERK, p-CREB, BDNF, BCL, and BAX levels, as well as neurite outgrowth (TUBB3 staining), were measured. (b) p-PKA, p-CaMKII, and p-ERK protein levels determined by immunoblot using GAPDH as a loading control (*n* = 3). To normalize, the protein expression level in untreated cells was set as 100%. (c) Measurements of neurite outgrowth (length, process, and branch) (*n* = 3). *p* values: induced vs. uninduced cells (^##^*p* < 0.01 and ^###^*p* < 0.001), LM-021-treated vs. untreated cells (^∗^*p* < 0.05 and ^∗∗^*p* < 0.01), or kinase inhibitor-treated vs. untreated cells (^&^*p* < 0.05, ^&&^*p* < 0.01, and ^&&&^*p* < 0.001) (one-way ANOVA with a post hoc Tukey test).

Tet-On ∆K280 tau_RD_-DsRed SH-SY5Y cells were also used to explore the CREB-mediated neuroprotective potential of LM-021 (Figure 7(a)). As shown in Figure 7(b), neither ∆K280 tau_RD_-DsRed expression nor LM-021 treatment significantly altered the level of PKA, CaMKII, or ERK. The induced expression of ∆K280 tau_RD_-DsRed caused decreased levels of p-PKA, p-CaMKII, and p-ERK, although not significantly, whereas treatment with LM-021 increased p-PKA, p-CaMKII, and p-ERK. In contrast, H-89 treatment reduced the upregulation of p-PKA, KN-62 treatment diminished the increase of p-CaMKII, and U0126 treatment attenuated the rescue of p-ERK.

Moreover, the induction of ∆K280 tau_RD_-DsRed expression resulted in reduced p-CREB, CREB, pro-BDNF, m-BDNF, and BCL2 and elevated BAX protein levels. Treatment with LM-021 significantly upregulated protein levels of p-CREB, CREB, pro-BDNF, m-BDNF, and BCL2 and decreased BAX protein level. In contrast, H-89, KN-62, and U0126 treatment attenuated the upregulation of p-CREB, pro-BDNF, m-BDNF, and BCL2 and reversed the reduction in BAX (Figure S7A). In ∆K280 tau_RD_-DsRed-expressing SH-SY5Y cells, LM-021 ameliorated the defects in neurite length and branch length, whereas H-89, KN-62, or U0126 treatment reversed these effects (Figure 7(c) and Figure S7B).

### 3.8. Pharmacokinetics of LM-021

Pharmacokinetic data for LM-021 were obtained by noncompartmental analysis using Phoenix WinNonlin. The calculated PK parameters in mice are summarized in Table 1. The elimination half-life (*t*_1/2_) of LM-021 in plasma and brain was 2.54 ± 0.79 and 2.17 ± 0.67 h, respectively, suggesting that after 5 half-lives, 97% of LM-021 in plasma/brain will be eliminated. The mean resident time (MRT) of LM-021 was 3.54 ± 0.93 h, indicating the average time that LM-021 molecules are present in the body before being eliminated. The plasma clearance of LM-021 was 1.85 ± 0.12 ml/min/kg, which is about 0.5% of the cardiac output blood flow of mice (400 ml/min/kg), indicating low clearance in mice. The total apparent volume of distribution at steady state (*V*_ss_) was 0.39 ± 0.08 l/kg, which is higher than the total plasma volume (0.05 l/kg) of mice, suggesting that LM-021 can distribute into tissues. After intravenous bolus injection, the systemic exposure (AUC_0-∞_) of LM-021 in plasma and brain was 45.28 ± 2.83 and 2.38 ± 0.03 *μ*g h/ml, respectively. LM-021 had a brain to plasma ratio of 5.3% in mice.

## 4. Discussion

Effective treatments to slow AD neurodegeneration are still unavailable. Lines of evidence have suggested that the CREB signaling pathway is a potential therapeutic target for AD. The decreased levels of phosphorylated CaMKII, ERK, and CREB have been shown in the hippocampus of old rats with impaired spatial memory [43–45]. Oligomeric A*β* treatment significantly downregulates CREB phosphorylation [16]. Accumulation of tau may cause synapse and memory impairment by inactivating CREB signaling and CaMKIV activity [46]. Here, we showed that the novel synthetic coumarin-chalcone derivative LM-021 activates CREB signaling. LM-021 provides neuroprotective effects through increasing phosphorylation of PKA, CaMKII, and ERK to activate CREB and enhancing the expression of its downstream genes in A*β*-GFP- and *Δ*K280 tau_RD_-DsRed-expressing SH-SY5Y cells. When A*β* and tau models were compared, the less reduction changes of phosphorylated PKA, CaMKII, and ERK in tau cells may explain that the effects of LM-021 on tau cells seem to be less significant than on A*β* cells.

The activation of CREB and expression of its downstream genes are regulated by protein kinases under the control of cAMP [10] or Ca^2+^ [47, 48]. CREB phosphorylation and nuclear translocation are largely mediated by Ca^2+^ influx, the mobilization of calmodulin to the nucleus, and activation of CaMKII and CaMKIV [49–51]. To facilitate CREB phosphorylation in our CRE-GFP 293 reporter cells, we treated the reporter cells with a Ca^2+^ ionophore to increase cytosolic Ca^2+^. We observed that CRE-motif-driven GFP fluorescence and CREB phosphorylation were augmented by the Ca^2+^ ionophore (Figures 2 and 5). These results are similar to those of other studies in PC12 cells and auditory neurons [52, 53]. The ability of LM-021 to activate the CREB signaling pathway was also demonstrated in these reporter cells. Of note, LM-021 also upregulated the expression of CREB in A*β*-GFP- and *Δ*K280 tau_RD_-DsRed-expressing SH-SY5Y cells (Figures S6A and S7A), further providing its neuroprotective effects by modulating the CREB signaling pathway.

To further understand the mechanism underlying the effect of LM-021 on CREB phosphorylation, we treated CRE-GFP reporter cells with H-89, KN-62, U0126, and wortmannin, which are inhibitors of PKA, CaMKII, ERK, and PI3K. The LM-021-increased phosphorylation of CREB and CRE-motif-driven GFP fluorescence were attenuated by the inhibition of PKA, CaMKII, and ERK, but not PI3K (Figure 5). In A*β*-GFP- and *Δ*K280 tau_RD_-DsRed-expressing SH-SY5Y cells, the reduced phosphorylation of PKA, CaMKII, ERK, and CREB indicates that these signaling pathways were all compromised by the overexpression of A*β* or tau protein (Figures 6 and 7). LM-021 improved neurite outgrowth and consistently increased the phosphorylation of PKA, CaMKII, and ERK, all of which in turn phosphorylate CREB and drive the expression of BDNF and BCL2. The decrease in CREB phosphorylation by inhibitors of PKA, CaMKII, and ERK counteracted the neuroprotective effects of LM-021. These results indicate that LM-021, working as a CREB enhancer by regulating the PKA, CaMKII, and ERK pathways, displays neuroprotective effects against A*β* and tau aggregation.

PKA is the main regulator of most cAMP-dependent physiological processes. The association of cAMP with a specific site on the surface of PKA leads to the release of catalytic subunits that phosphorylate downstream substrates [54]. PKA can remodel neurite outgrowth in developing neurons. Decreased PKA activity by adenylyl cyclase inhibitors results in deficient neurite outgrowth, while enhanced PKA activity by forskolin or dibutyryl-cAMP promotes neurite outgrowth [55]. In our study, the reduction in PKA activity caused by the specific inhibitor H-89 significantly counteracted the improved neurite outgrowth induced by LM-021. This observation is consistent with the reported neuritogenic activity mediated by cAMP/PKA in human SH-SY5Y cells [56]. On the other hand, while a basal level of PKA activity is required to promote neurite outgrowth, a high level of PKA activity shortens and simplifies neurites [57]. Thus, a proper level of PKA activity is critical for promoting optimal neurite growth.

CaMKII is regulated by the Ca^2+^/calmodulin complex [58]. Postmortem brain analyses show that CaMKII-expressing neurons are selectively lost in the hippocampus of severe AD patients [59, 60]. The phosphorylation of CaMKII is also reduced in the hippocampus of patients with mild cognitive impairment or severe AD [61, 62]. Upregulation of CaMKII phosphorylation by spatial training can improve spatial learning and memory in an *APP* transgenic mouse model of AD [63]. In A*β*-GFP- and *Δ*K280 tau_RD_-DsRed-expressing cells, we consistently observed that a decrease in CaMKII phosphorylation by KN-62 diminished the neuroprotective effects of LM-021, suggesting a key role for CaMKII in the mechanism underlying AD neurodegeneration.

ERK is highly expressed in the central nervous system, and the ERK pathway plays multiple roles in the activity-dependent regulation of neuron function [64, 65]. ERK activity controls the proliferation and differentiation of neurons and glia during brain development [66, 67]. Consistent with our findings, the phosphorylation of ERK by *α*-lipoic acid has been reported to promote neurite outgrowth [68]. Evidence suggests that ERK activity is markedly increased in AD [69–71]. ERK is significantly activated in *APP* transgenic mice [72], and tau can be hyperphosphorylated by ERK [73]. ERK colocalises with neurofibrillary tangles in neurons and with tau in subcellular compartments [74]. More details regarding the function of ERK in pathological conditions may provide insight into the pathogenesis of AD.

CREB phosphorylation enhances the expression of CRE-mediated genes, including BCL2 and BDNF, which may provide neuroprotection against apoptosis [75, 76]. BCL2 inhibits the BAX activation and prevents it from translocation to the mitochondria, thereby decreasing apoptosis; conversely, downregulated BCL2 promotes mitochondrial BAX homooligomerization, resulting in subsequent induction of the cytochrome c-mediated apoptotic cascade [77–79]. In AD, A*β* induces the downregulation of BCL2 and upregulation of BAX [80]. The ratio of BCL2 to BAX has been shown to correlate negatively with the level of tau phosphorylation [81]. As an important member of the classic neurotrophin family of growth factors, BDNF promotes neuron survival, differentiation, and plasticity [82]. In SH-SY5Y cells, treatment with oligomeric A*β* significantly causes downregulation of BDNF expression [83]. Tau overexpression or hyperphosphorylation reduces BDNF expression in primary neurons and tau transgenic mice [84, 85]. We also consistently observed that overexpression of A*β*-GFP or *Δ*K280 tau_RD_-DsRed reduces BCL2 and BDNF and increases BAX expression. In this study, we show that LM-021 normalizes the expression of these CREB-responsive genes.

LM-021 displays good oral bioavailability, BBB penetration capability (Figure 1), and low toxicity in SH-SY5Y cells (Figures 3 and 4). Our pharmacokinetic study showed that LM-021 has a low clearance and a brain to plasma ratio of 5.3% in mice, which allows LM-021 to distribute into tissues, including the brain. Although 5.3% is a modest brain to plasma ratio, some of the drugs used to treat neurodegenerative diseases have an even lower ratio. For example, without an enzyme inhibitor, only about 1% of orally administered levodopa enters the brain [86]. Therefore, LM-021 shows the potential to cross the BBB and may be a suitable candidate compound for treating AD. LM-021 also demonstrated significant inhibitory effects on aggregation and ROS reduction in both A*β*-GFP- and *Δ*K280 tau_RD_-DsRed-expressing SH-SY5Y cells. As A*β* and tau pathologies are likely synergistic, the observation that LM-021 targets both of these proteins suggests that it may be particularly suitable for use in pleiotropic treatments for AD. LM-021 exhibited greater inhibitory effects against A*β* and tau aggregation than did curcumin or Congo red, indicating its strong potential as a therapeutic agent. Furthermore, LM-021 displayed neuroprotective effects not only by rescuing neurite outgrowth deficits but also by reducing caspase 1 activity in A*β*-GFP- and *Δ*K280 tau_RD_-DsRed-expressing SH-SY5Y cells. Given that tau accumulations are commonly seen in other neurodegenerative diseases, including frontotemporal dementia, progressive supranuclear palsy, Pick's disease, and corticobasal syndrome, we expect that LM-021 may be promising for treating these tauopathies as well.

To determine whether the coumarin-chalcone derivatives have chemical chaperone activity, we used the Thioflavin T assay to assess misfolding of aggregated synthetic A*β*_42_ and *E. coli*-derived *Δ*K280 tau_RD_. The results show that the coumarin-chalcone derivative LM-021 directly hinders A*β*_42_ and tau aggregation (Figure 1). Whether the upregulation of CREB phosphorylation and its upstream pathways PKA, CaMKII, and ERK occurs subsequent to aggregate inhibition or is directly enhanced by LM-021, or both, necessitates further studies.

Finally, preconditioning signal leading to cellular protection through an important redox reaction has the hormesis feature, which can ameliorate aging associated with free radical accumulation and inflammatory responses involved in neurodegenerative/neuroprotective mechanisms [87]. Several reports have shown the relationship between polyphenol compounds, redox status, and the vitagene network and its possible biological relevance in neuroprotection [88, 89]. The tested novel compound LM-021 displays significant antioxidative and neuroprotection effects and probably implicates its role as an agent involved in the vitagene network. Although our study has shown the maximal effective dose of inhibiting aggregation and neuroprotection effects among 3 different doses, we have not yet determined if higher concentrations induce significant cell toxicity and display less neuroprotection effect. Further studies will be demanded to explore optimised doses of LM-021 in treating and preventing AD, based on the hormesis feature of antioxidant.

## 5. Conclusions

In the present study, we show that the CREB signaling pathway in A*β*-GFP- and *Δ*K280 tau_RD_-DsRed-expressing SH-SY5Y cells is compromised, which leads to decreased BCL2 and BDNF, elevated BAX and oxidative stress, and subsequent neurite outgrowth deficits. LM-021 exerts neuroprotective effects in these cells by regulating the PKA, CaMKII, and ERK pathways, all of which subsequently increase CREB phosphorylation and neurite outgrowth. Given that multiple pathways are involved in AD pathogenesis, the targeting of multiple pathways by LM-021 to provide neuroprotection may be particularly promising in the development of drugs for treating AD. The neuroprotective effects of LM-021 observed in the tau cell model also suggest this agent as a novel candidate for treating other neurodegenerative tauopathies. The demonstration that LM-021 has good oral availability and BBB penetration suggests its use for targeting neurons in AD patients by oral administration. Nevertheless, it is important to note that the effects of LM-021 were only observed in cell models. Future studies in animal models are necessary to confirm these results before investigating its therapeutic application to clinical trials.

## Figures and Tables

**Table 1:** Summary of PK parameters for LM-021 in ICR male mice.

Sample type	C_0_ (ng/ml)	*t*_1/2_ (h)	MRT (h)	AUC_(0-∞)_ (ng·h/ml)^a^	CL (ml/min/kg)	*V*_ss_ (l/kg)	Brain/plasma ratio^b^
Plasma	20375 ± 9475	2.54 ± 0.79	3.54 ± 0.93	45278 ± 2834	1.85 ± 0.12	0.39 ± 0.08	0.053
Brain^a^	NA	2.17 ± 0.67	NA	2379 ± 34	NA	NA	

^a^The brain tissue density was assumed to be 1 g/ml. ^b^Brain-plasma ratio was calculated by the AUC_(0-∞)_ ratios. NA: not applicable; C_0_: concentration at time zero; *t*_1/2_: elimination half-life; MRT: mean residence time; AUC_(0-∞)_: the area under the concentration-time curve from time 0 extrapolated to infinity; CL: body clearance; *V*_ss_: an estimate of the volume of distribution at steady state.

## Data Availability

The data used to support the findings of this study are available from the corresponding author upon request.

## Supplementary Materials

Supplementary Materials Preparation and analysis of mouse plasma and brain homogenate samples. Figure S1: oral bioavailability prediction and TEM examination of A*β* and tau aggregates. Figure S2: nucleotide sequence of synthetic CRE fused to TATA-like promoter and CRE fluorescence reporter assay. Figure S3: dose-response curves based on GFP fluorescence and ROS images in A*β*-GFP cells. Figure S4: dose-response curves based on DsRed fluorescence and ROS images in *Δ*K280 tau_RD_-DsRed cells. Figure S5: experimental flow chart to examine LM-021-mediated kinase activation. Figure S6: regulation of CREB signaling pathway and neurite outgrowth images in A*β*-GFP cells. Figure S7: regulation of CREB signaling pathway and neurite outgrowth images in *Δ*K280 tau_RD_-DsRed cells.
