# Supplementary material for: Novel Synthetic Coumarin-Chalcone Derivative (E)-3-(3-(4-(Dimethylamino)Phenyl)Acryloyl)-4-Hydroxy-2H-Chromen-2-One Activates CREB-Mediated Neuroprotection in Aβ and Tau Cell Models of Alzheimer's Disease
Source: Oxid Med Cell Longev. 2021 Nov 13;2021:3058861. doi: 10.1155/2021/3058861 (PMC8605905; doi:10.1155/2021/3058861)
Supplement: Supplementary Materials — Preparation and analysis of mouse plasma and brain homogenate samples. Figure S1: oral bioavailability prediction and TEM examination of Aβ and tau aggregates. Figure S2: nucleotide sequence of synthetic CRE fused to TATA-like promoter and CRE fluorescence reporter assay. Figure S3: dose-response curves based on GFP fluorescence and ROS images in Aβ-GFP cells. Figure S4: dose-response curves based on DsRed fluorescence and ROS images in ΔK280 tauRD-DsRed cells. Figure S5: experimental flow chart to examine LM-021-mediated kinase activation. Figure S6: regulation of CREB signaling pathway and neurite outgrowth images in Aβ-GFP cells. Figure S7: regulation of CREB signaling pathway and neurite outgrowth images in ΔK280 tauRD-DsRed cells. [file 3058861.f1.zip › 3058861.f1/4_14 Supplementary Materials.pdf]

Supplementary Description

Preparation and analysis of mouse plasma and brain homogenate samples. A solvent-based protein precipitation procedure was used for analyzing plasma and brain homogenate samples. A 20-μl aliquot of each plasma sample was mixed with 60 μl of acetonitrile. For brain samples, collected brains were homogenized in 3 volumes of methanol, and then 50 μl of brain homogenate was mixed with 50 μl of acetonitrile. After vortexing and centrifugation (20,000 × g for 5 min), the extracted supernatants were collected. Liquid chromatography – tandem mass spectrometry (LC-MS/MS) analyses was performed using an Agilent 1200 (Agilent Technologies, Palo Alto, CA, USA) coupled to a QTrap 5500 triple-quadrupole mass spectrometer (AB SCIEX, Foster City, CA, USA). Chromatographic separation was performed on an Eclipse Plus C18 column (3.5 μm; 4.6 × 50 mm; Agilent Technologies) with the mobile phase performed at a constant flow rate of 1.0 ml/min using a binary solvent system: solvent A, 5 mM ammonium acetate; solvent B, acetonitrile. The transition (precursor ion to daughter ion) monitored was m/z 336.2 → 321.0 for LM-021. Multiple reaction monitoring (MRM) data were acquired, and the chromatograms were integrated using Analyst 1.6.3 software (Applied Biosystems). A weighted (1/x) linear regression was used to generate the calibration curves from standards and to calculate the sample concentrations. The calibration curve was linear over the concentration range of 1 to 200 ng/ml.

Figure S1: Oral bioavailability prediction and TEM examination of Aβ and Tau aggregates. (A) Molecular weight (MW), hydrogen bond donor (HBD), hydrogen bond acceptor (HBA), calculated octanol-water partition coefficient (cLogP), polar surface area (PSA), and predicted blood-brain barrier (BBB) score of these compounds. (B) Transmission electron microscope (TEM) examination of $A\beta_{42}$/ΔK280 tau_{RD} aggregation without or with curcumin/congo red and LM-021 (10 μM) addition.

Figure S2: Nucleotide sequence of synthetic CRE fused to TATA-like promoter and CRE fluorescence reporter assay. (A) Nucleotide sequence of synthetic fragment containing two copies of the CRE (cAMP response element) (blue box) fused to P_{TAL} (TATA-like promoter) region from the HSV-TK (herpes simplex virus thymidine kinase) gene (pink) flanked by *Mlu*I (ACGCGT) and *Age*I (ACCGGT) restriction sites (red). (B) Experimental flow chart for GFP fluorescence analysis of CRE reporter cells. CRE-GFP 293 cells were treated with tested compounds (5–10 μM) and Ca^{2+} ionophore (2 μM) for 5 h and GFP fluorescence assessed by flow cytometry.

Figure S3: Dose response curves based on GFP fluorescence and ROS images in Aβ-GFP cells. (A) Dose response curves of test compounds based on GFP fluorescence measurement. (B) Images of CellROX assay of Aβ-GFP cells uninduced, induced, or treated with test compounds at 5 μM. (C) Representative images of TUBB3 signal (yellow) in differentiated Aβ-GFP SH-SY5Y cells uninduced, induced or treated with test compounds (5 μM). Nuclei were counterstained with DAPI (blue). Also shown were segmented images with multi-coloured mask to outline outgrowth of individual cell body for neurite outgrowth quantification.

Figure S4: Dose response curves based on DsRed fluorescence and ROS images in ΔK280 tau$_{RD}$-DsRed cells. (A) Dose response curves of test compounds based on DsRed fluorescence measurement. (B) Images of DCF assay of ΔK280 tau$_{RD}$-DsRed cells uninduced, induced, or treated with test compounds at 10 μM. (C) Representative images of TUBB3 signal (green) in differentiated ΔK280 tau$_{RD}$-DsRed SH-SY5Y cells uninduced, induced, or treated with test compounds (10 μM). Nuclei were counterstained with DAPI (blue). Also shown were segmented images with multi-coloured mask to outline outgrowth of individual cell body for neurite outgrowth quantification.

Figure S5: Experimental flow chart to examine LM-021-mediated kinase activation. On day 2, CRE-GFP 293 cells were treated with kinase inhibitors (10 μM) for 4 h, followed by Ca^{2+} ionophore (2 μM) and compound addition (10 μM) for 5 h. GFP fluorescence and p-CREB and GFP levels were assessed.

Figure S6: Regulation of CREB signaling pathway and neurite outgrowth images in Aβ-GFP cells. (A) p-CREB, BDNF, BCL, and BAX protein levels analyzed by immunoblot using GAPDH as a loading control (n = 3). To normalize, protein expression level in untreated cells was set at 100%. p values: comparisons between induced vs. uninduced cells ($^{\#}$: $p < 0.05$, $^{\#\#}$: $p < 0.01$, $^{\#\#\#}$: $p < 0.001$), LM-021-treated vs. untreated cells (**: $p < 0.01$, ***: $p < 0.001$), or kinase inhibitor-treated vs. untreated cells ($^{\&}$: $p < 0.05$, $^{\&\&}$: $p < 0.01$, $^{\&\&\&}$: $p < 0.001$). (one-way ANOVA with a *post hoc* Tukey test) (B) Representative images of TUBB3 signal (yellow) and segmented images with multi-coloured mask to outline outgrowth of individual cell body. Nuclei were counterstained with DAPI (blue).

Figure S7: Regulation of CREB signaling pathway and neurite outgrowth images in ΔK280 tau$_{RD}$-DsRed cells. (A) p-CREB, BDNF, BCL, and BAX protein levels

analyzed by immunoblot using GAPDH as a loading control (*n* = 3). To normalize, protein expression level in untreated cells was set at 100%. *p* values: comparisons between induced vs. uninduced cells (#: $p < 0.05$, ###: $p < 0.001$), LM-021-treated vs. untreated cells (*: $p < 0.05$), or kinase inhibitor-treated vs. untreated cells (&: $p < 0.05$, &&: $p < 0.01$). (one-way ANOVA with a *post hoc* Tukey test) (B) Representative images of TUBB3 signal (green) and segmented images with multi-coloured mask to outline outgrowth of individual cell body. Nuclei were counterstained with DAPI (blue).
