# Supplementary material for: Novel Synthetic Coumarin-Chalcone Derivative (E)-3-(3-(4-(Dimethylamino)Phenyl)Acryloyl)-4-Hydroxy-2H-Chromen-2-One Activates CREB-Mediated Neuroprotection in Aβ and Tau Cell Models of Alzheimer's Disease
Source: Oxid Med Cell Longev. 2021 Nov 13;2021:3058861. doi: 10.1155/2021/3058861 (PMC8605905; doi:10.1155/2021/3058861)
Supplement: Supplementary Materials — Preparation and analysis of mouse plasma and brain homogenate samples. Figure S1: oral bioavailability prediction and TEM examination of Aβ and tau aggregates. Figure S2: nucleotide sequence of synthetic CRE fused to TATA-like promoter and CRE fluorescence reporter assay. Figure S3: dose-response curves based on GFP fluorescence and ROS images in Aβ-GFP cells. Figure S4: dose-response curves based on DsRed fluorescence and ROS images in ΔK280 tauRD-DsRed cells. Figure S5: experimental flow chart to examine LM-021-mediated kinase activation. Figure S6: regulation of CREB signaling pathway and neurite outgrowth images in Aβ-GFP cells. Figure S7: regulation of CREB signaling pathway and neurite outgrowth images in ΔK280 tauRD-DsRed cells. [file 3058861.f1.zip › 3058861.f1/Fig-S2.pdf]

A

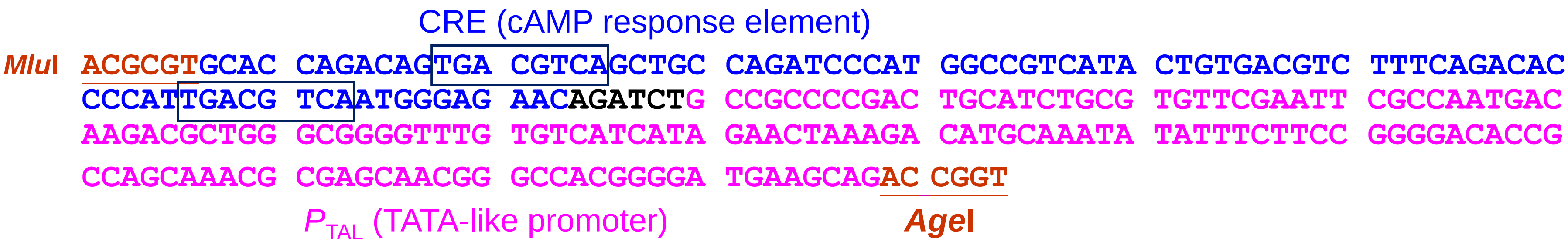
CRE (cAMP response element)
MluI
ACGCGTGCAC CAGACAGTGA CGTCAGCTGC CAGATCCCAT GGCCGTCATA CTGTGACGTC TTTCAGACAC
CCCATTTGACG TCAATGGGAG AACAGATCTG CCGCCCCGAC TGCATCTGCG TGTTCGAATT CGCCAATGAC
AAGACGCTGG GCGGGGTTTG TGTCATCATA GAACTAAAGA CATGCAAATA TATTTCTTCC GGGGACACCG
CCAGCAAACG CGAGCAACGG GCCACGGGGA TGAAGCAGAC CGGT
P TAL (TATA-like promoter)
AgeI

B

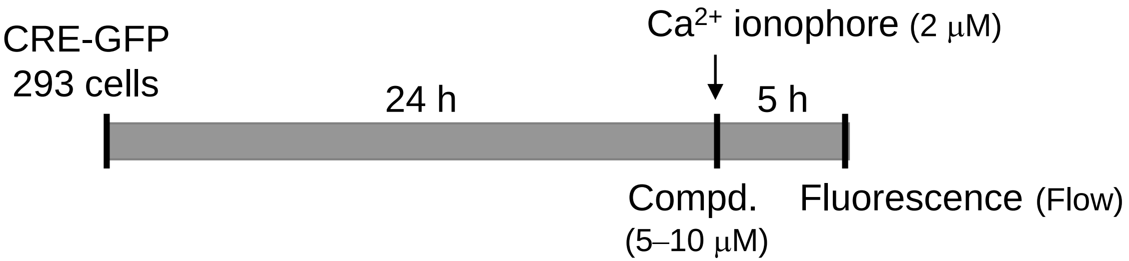
CRE-GFP
293 cells
24 h
Ca2+ ionophore (2 μM)
5 h
Compd.
(5–10 μM)
Fluorescence (Flow)
