# Supplementary figures and images for: Novel Synthetic Coumarin-Chalcone Derivative (E)-3-(3-(4-(Dimethylamino)Phenyl)Acryloyl)-4-Hydroxy-2H-Chromen-2-One Activates CREB-Mediated Neuroprotection in Aβ and Tau Cell Models of Alzheimer's Disease
Source: Oxid Med Cell Longev. 2021 Nov 13;2021:3058861. doi: 10.1155/2021/3058861 (PMC8605905; doi:10.1155/2021/3058861)

**A**

| Compound | MW     | HBD | HBA | cLogP | PSA  | BBB score<br>(Threshold: 0.02) |
|----------|--------|-----|-----|-------|------|--------------------------------|
| LM-016   | 306.31 | 1   | 4   | 4.45  | 63.6 | 0.145                          |
| LM-021   | 335.35 | 1   | 5   | 4.11  | 66.8 | 0.098                          |
| LM-022   | 282.25 | 1   | 5   | 3.12  | 72.8 | 0.153                          |

**B**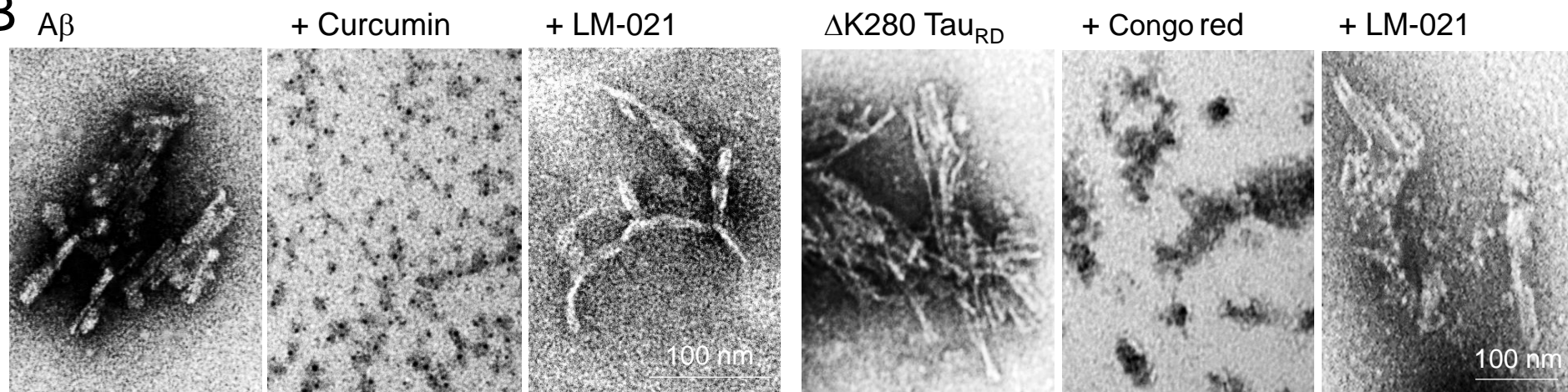

Supplement: Supplementary Materials — Preparation and analysis of mouse plasma and brain homogenate samples. Figure S1: oral bioavailability prediction and TEM examination of Aβ and tau aggregates. Figure S2: nucleotide sequence of synthetic CRE fused to TATA-like promoter and CRE fluorescence reporter assay. Figure S3: dose-response curves based on GFP fluorescence and ROS images in Aβ-GFP cells. Figure S4: dose-response curves based on DsRed fluorescence and ROS images in ΔK280 tauRD-DsRed cells. Figure S5: experimental flow chart to examine LM-021-mediated kinase activation. Figure S6: regulation of CREB signaling pathway and neurite outgrowth images in Aβ-GFP cells. Figure S7: regulation of CREB signaling pathway and neurite outgrowth images in ΔK280 tauRD-DsRed cells. [file 3058861.f1.zip › 3058861.f1/Fig-S1.pdf]

A

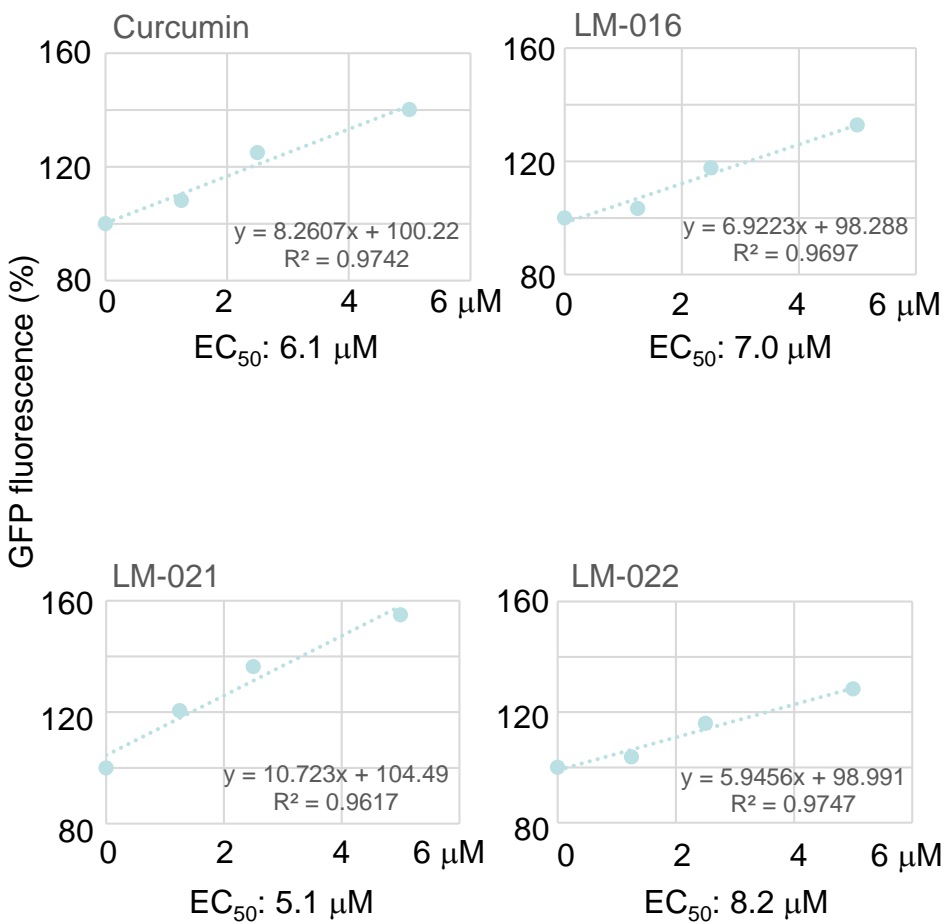

B CellROX/Phase

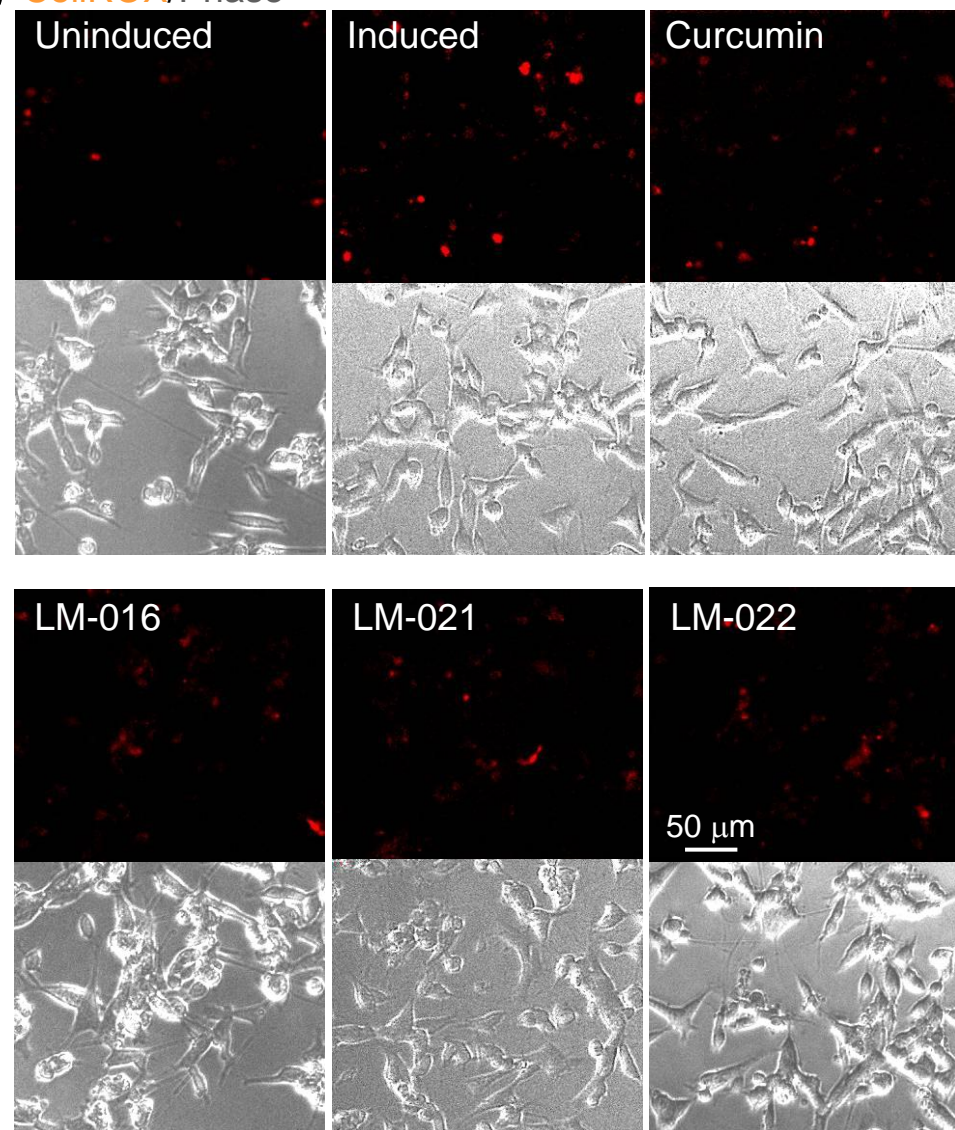

C

TUBB3/DAPI

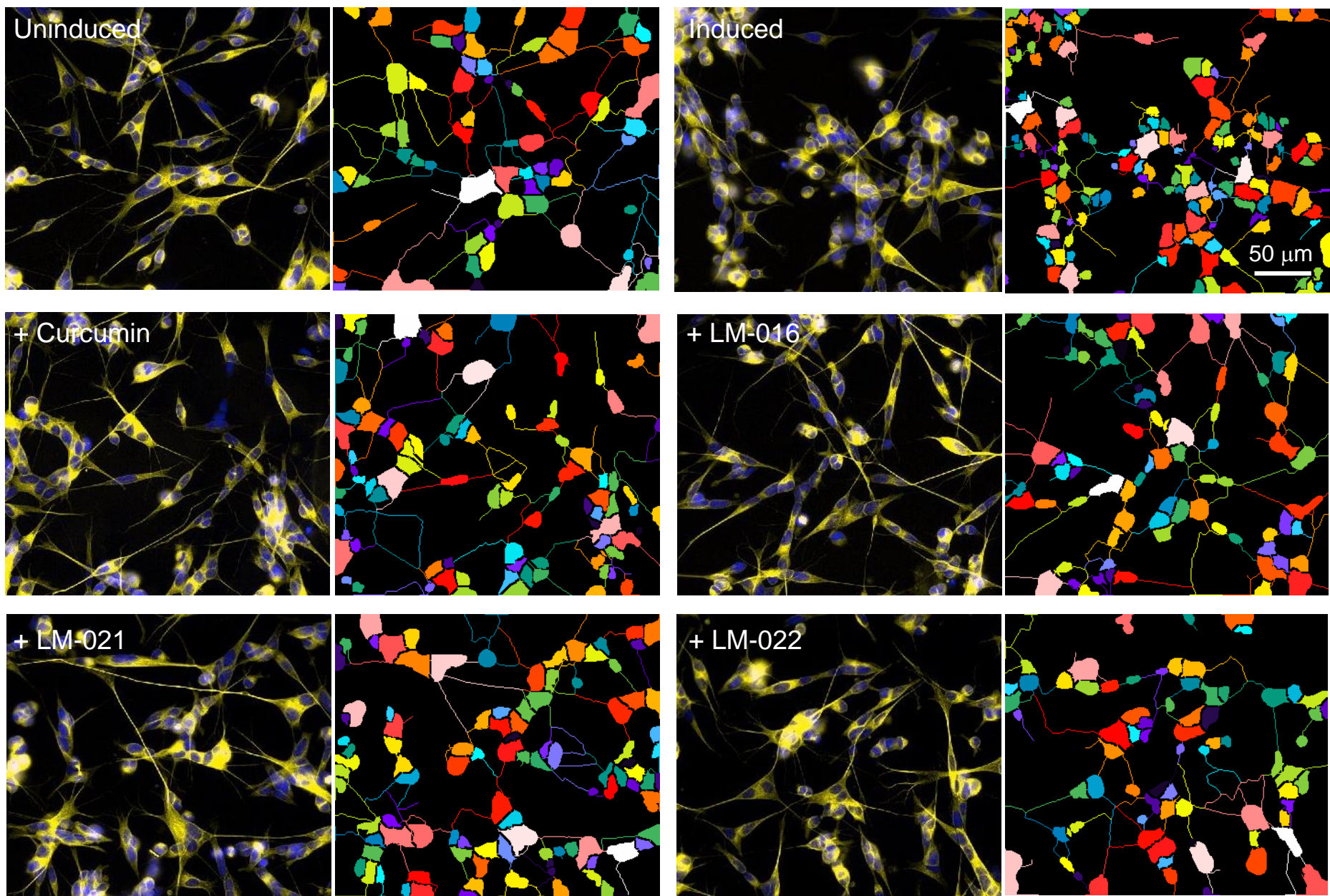

Supplement: Supplementary Materials — Preparation and analysis of mouse plasma and brain homogenate samples. Figure S1: oral bioavailability prediction and TEM examination of Aβ and tau aggregates. Figure S2: nucleotide sequence of synthetic CRE fused to TATA-like promoter and CRE fluorescence reporter assay. Figure S3: dose-response curves based on GFP fluorescence and ROS images in Aβ-GFP cells. Figure S4: dose-response curves based on DsRed fluorescence and ROS images in ΔK280 tauRD-DsRed cells. Figure S5: experimental flow chart to examine LM-021-mediated kinase activation. Figure S6: regulation of CREB signaling pathway and neurite outgrowth images in Aβ-GFP cells. Figure S7: regulation of CREB signaling pathway and neurite outgrowth images in ΔK280 tauRD-DsRed cells. [file 3058861.f1.zip › 3058861.f1/Fig-S3.pdf]

A

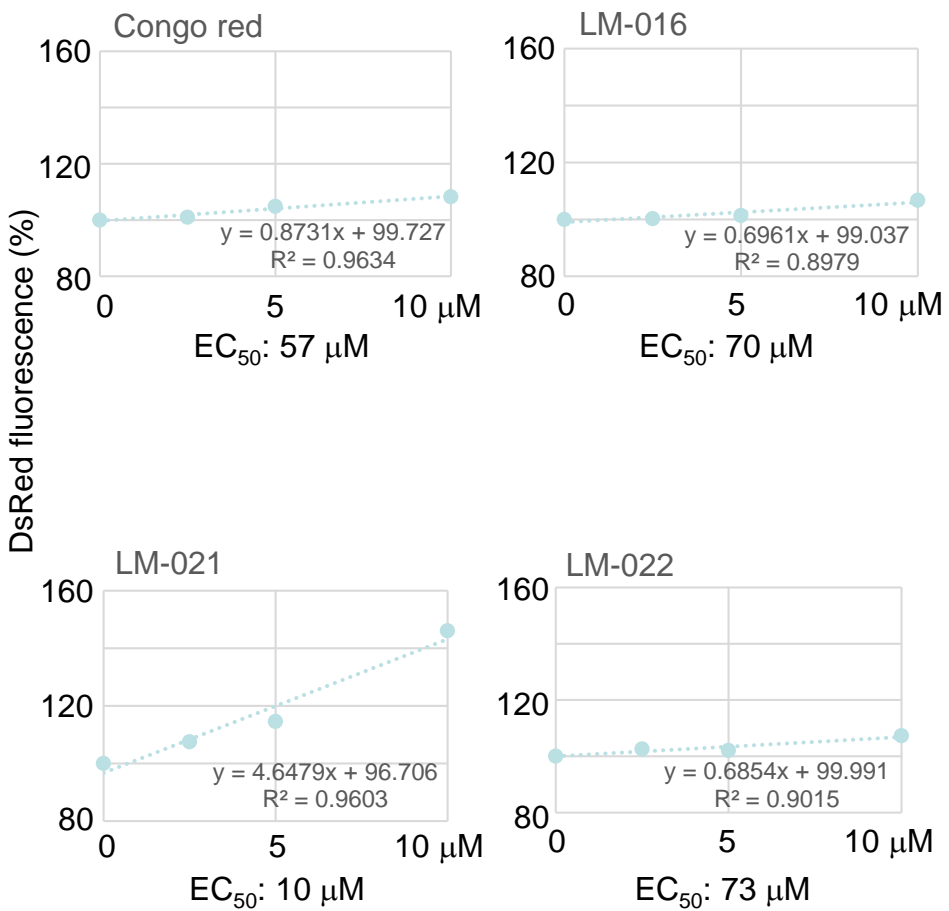

B

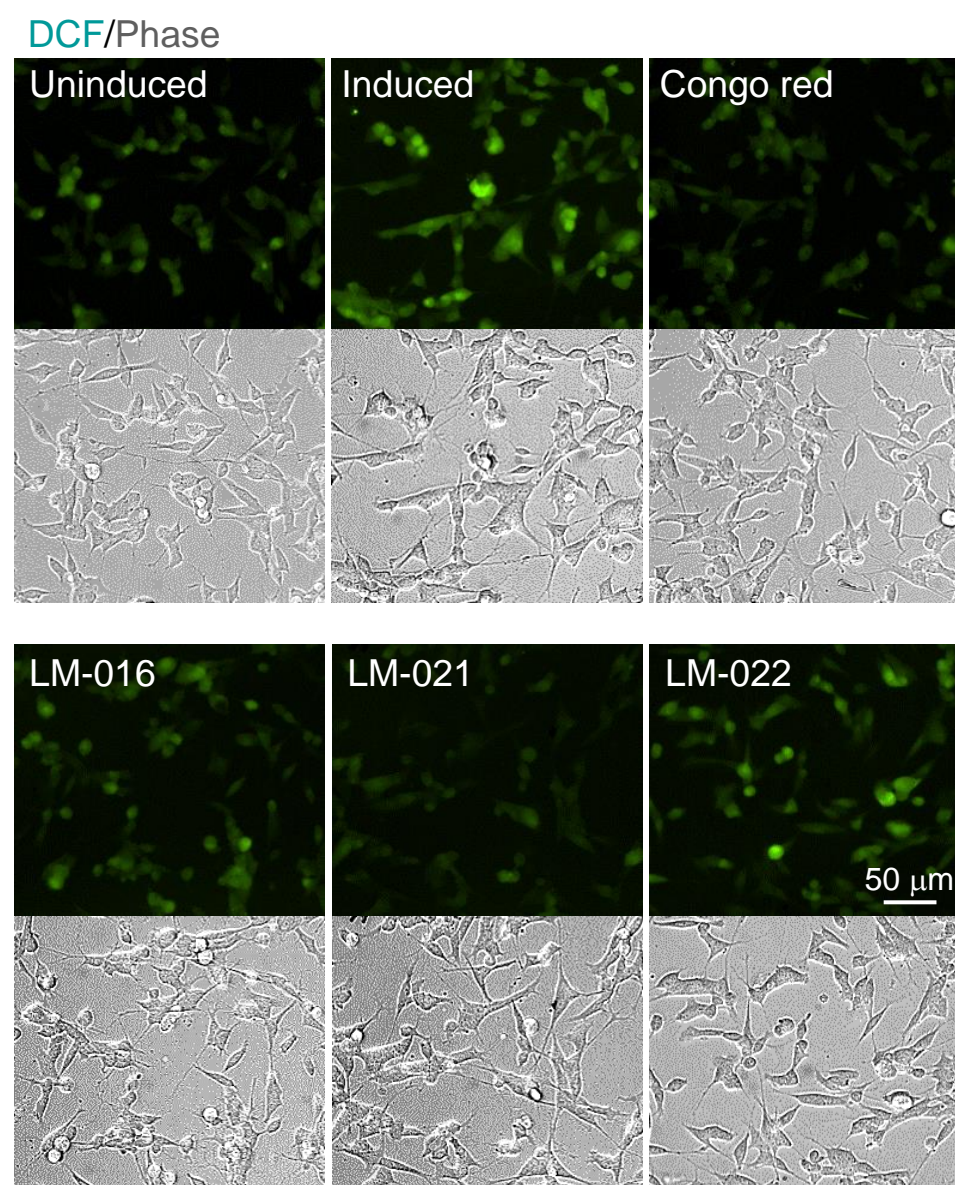

C

TUBB3/DAPI

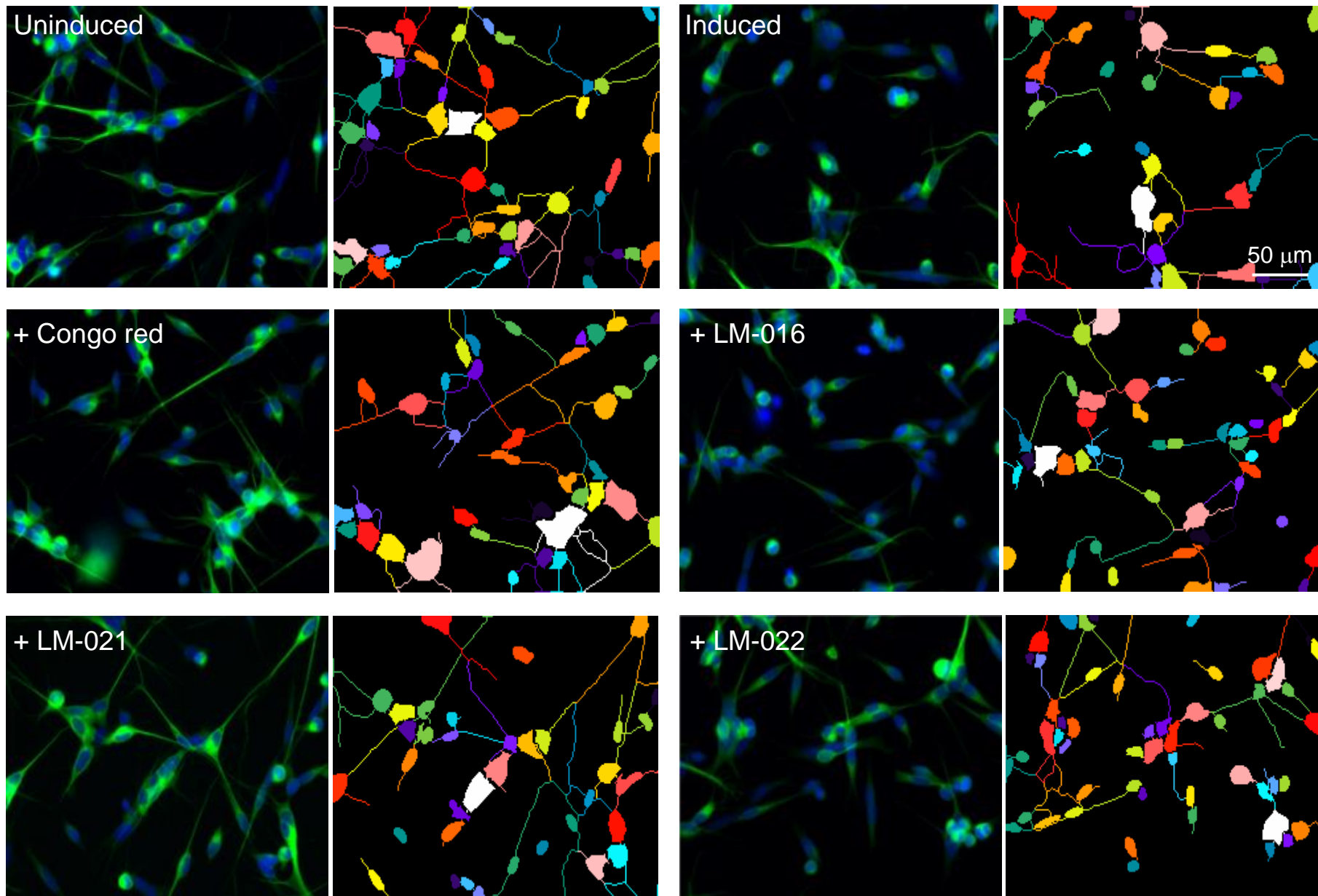

Supplement: Supplementary Materials — Preparation and analysis of mouse plasma and brain homogenate samples. Figure S1: oral bioavailability prediction and TEM examination of Aβ and tau aggregates. Figure S2: nucleotide sequence of synthetic CRE fused to TATA-like promoter and CRE fluorescence reporter assay. Figure S3: dose-response curves based on GFP fluorescence and ROS images in Aβ-GFP cells. Figure S4: dose-response curves based on DsRed fluorescence and ROS images in ΔK280 tauRD-DsRed cells. Figure S5: experimental flow chart to examine LM-021-mediated kinase activation. Figure S6: regulation of CREB signaling pathway and neurite outgrowth images in Aβ-GFP cells. Figure S7: regulation of CREB signaling pathway and neurite outgrowth images in ΔK280 tauRD-DsRed cells. [file 3058861.f1.zip › 3058861.f1/Fig-S4.pdf]

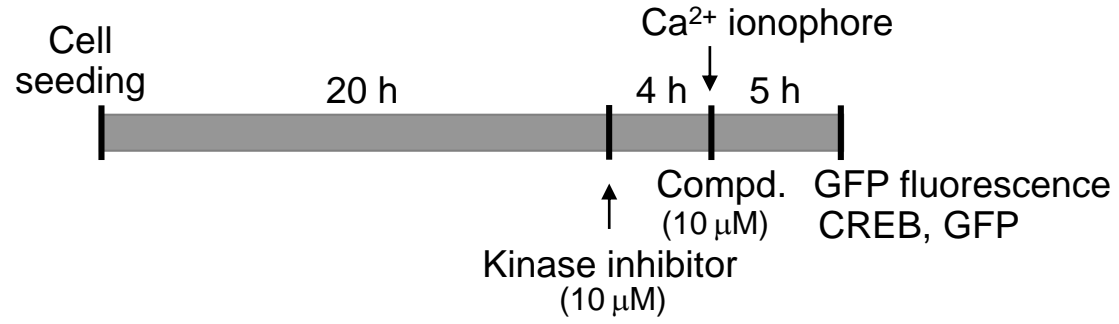

Supplement: Supplementary Materials — Preparation and analysis of mouse plasma and brain homogenate samples. Figure S1: oral bioavailability prediction and TEM examination of Aβ and tau aggregates. Figure S2: nucleotide sequence of synthetic CRE fused to TATA-like promoter and CRE fluorescence reporter assay. Figure S3: dose-response curves based on GFP fluorescence and ROS images in Aβ-GFP cells. Figure S4: dose-response curves based on DsRed fluorescence and ROS images in ΔK280 tauRD-DsRed cells. Figure S5: experimental flow chart to examine LM-021-mediated kinase activation. Figure S6: regulation of CREB signaling pathway and neurite outgrowth images in Aβ-GFP cells. Figure S7: regulation of CREB signaling pathway and neurite outgrowth images in ΔK280 tauRD-DsRed cells. [file 3058861.f1.zip › 3058861.f1/Fig-S5.pdf]

**A**

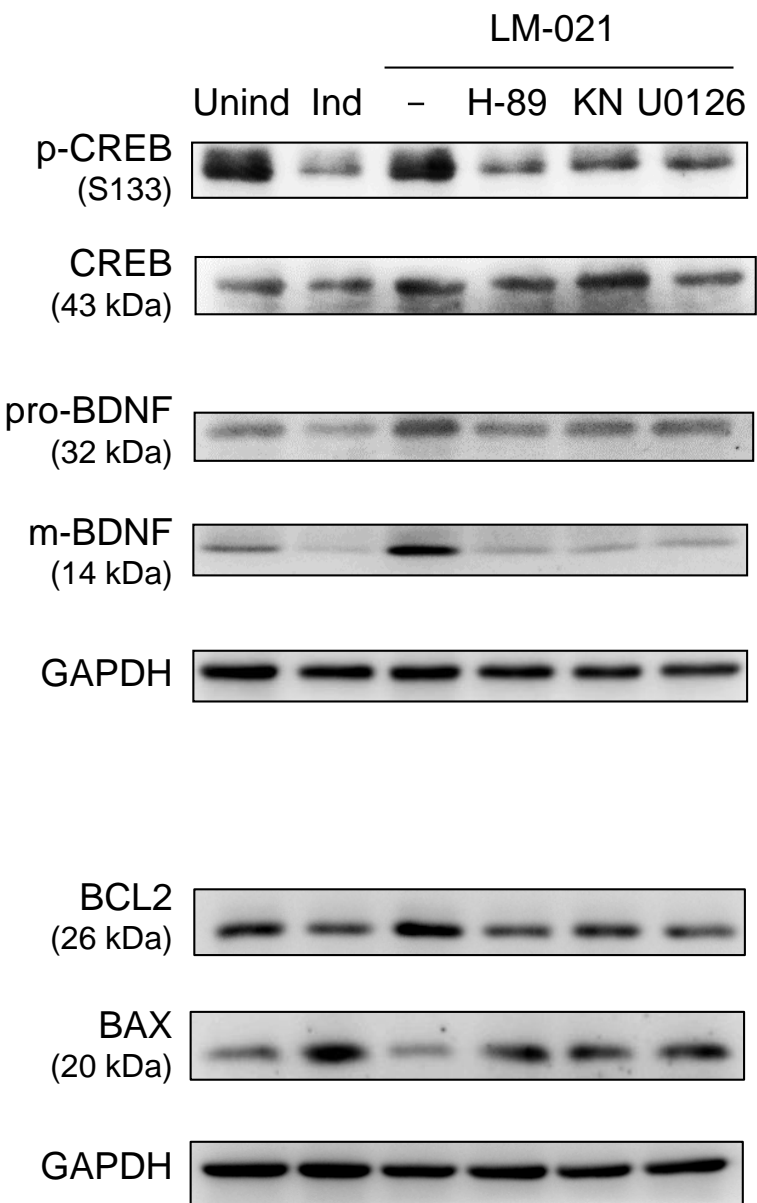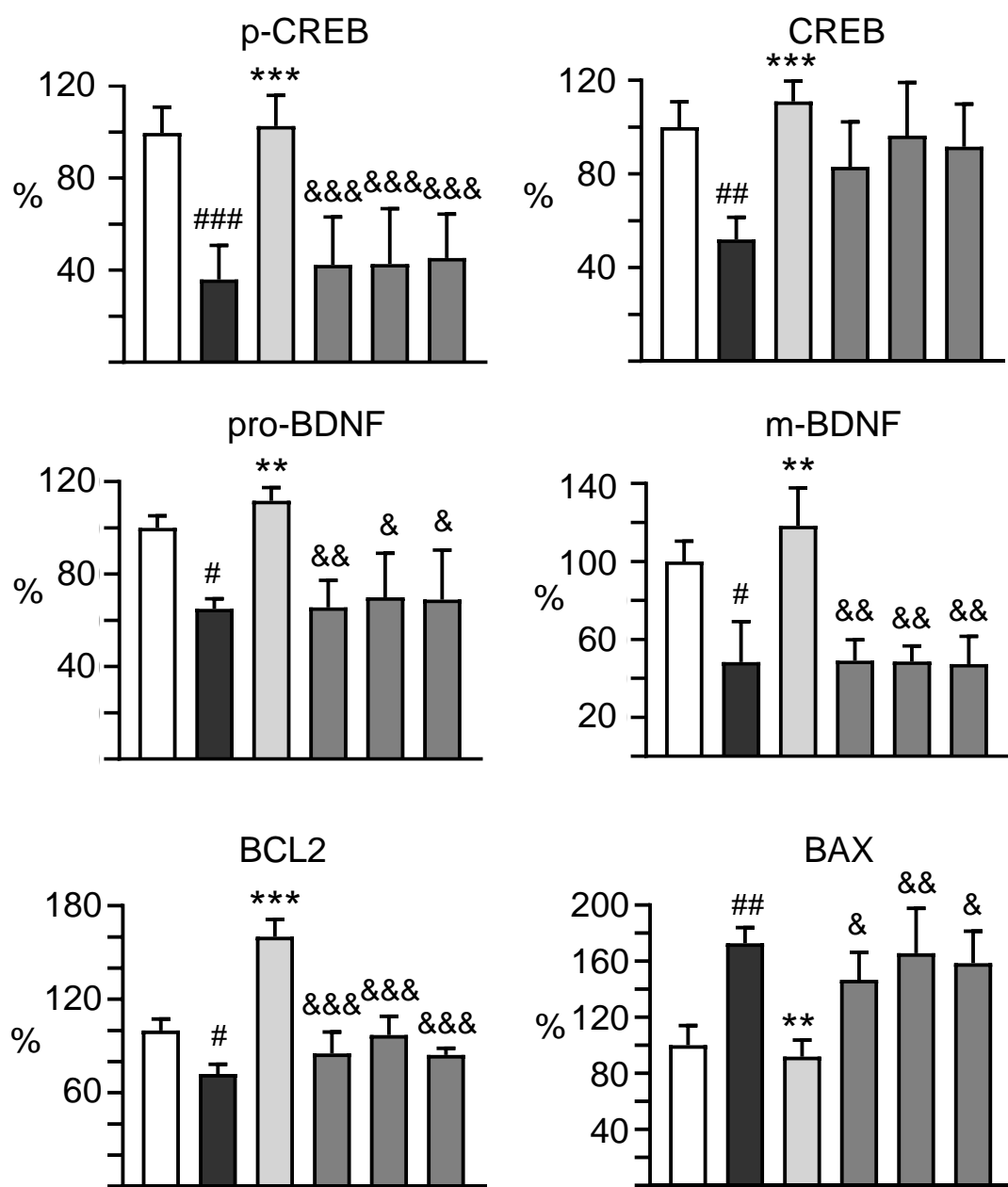

**B****TUBB3/DAPI**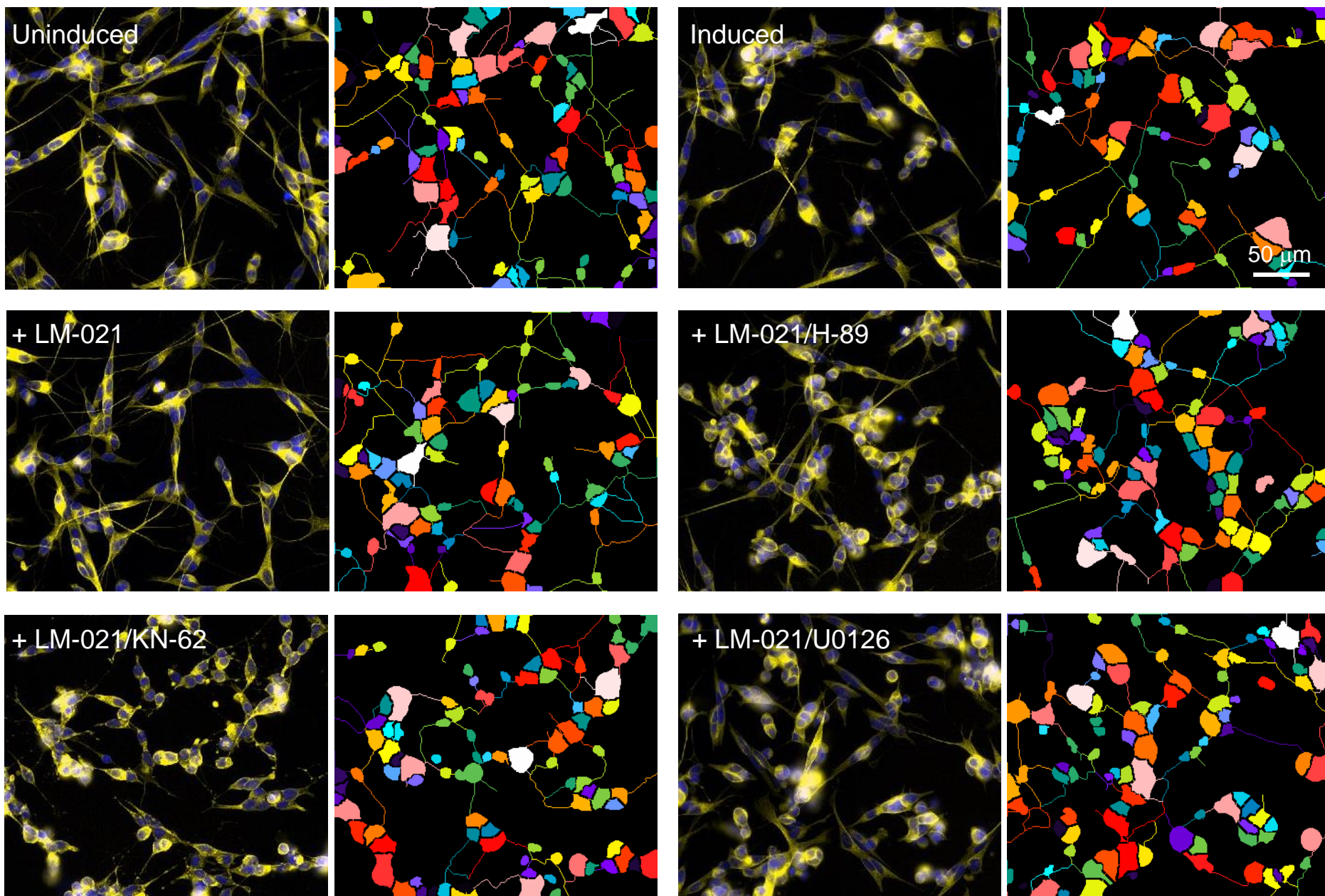

Supplement: Supplementary Materials — Preparation and analysis of mouse plasma and brain homogenate samples. Figure S1: oral bioavailability prediction and TEM examination of Aβ and tau aggregates. Figure S2: nucleotide sequence of synthetic CRE fused to TATA-like promoter and CRE fluorescence reporter assay. Figure S3: dose-response curves based on GFP fluorescence and ROS images in Aβ-GFP cells. Figure S4: dose-response curves based on DsRed fluorescence and ROS images in ΔK280 tauRD-DsRed cells. Figure S5: experimental flow chart to examine LM-021-mediated kinase activation. Figure S6: regulation of CREB signaling pathway and neurite outgrowth images in Aβ-GFP cells. Figure S7: regulation of CREB signaling pathway and neurite outgrowth images in ΔK280 tauRD-DsRed cells. [file 3058861.f1.zip › 3058861.f1/Fig-S6.pdf]

A

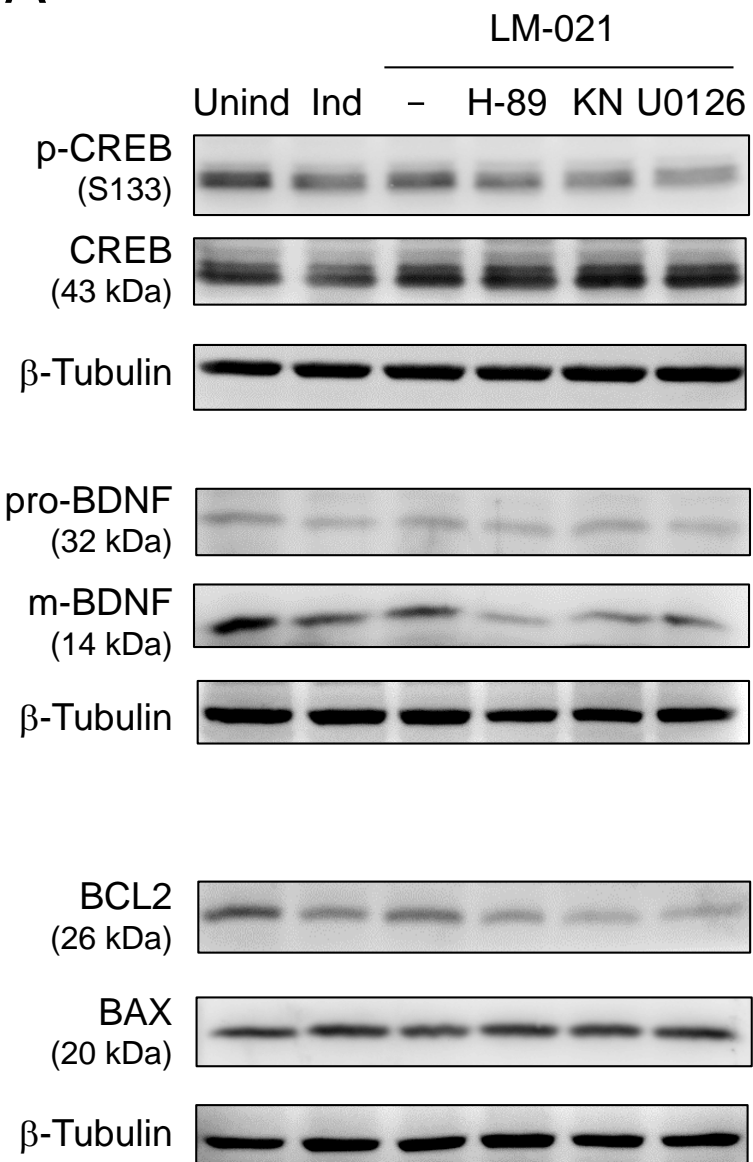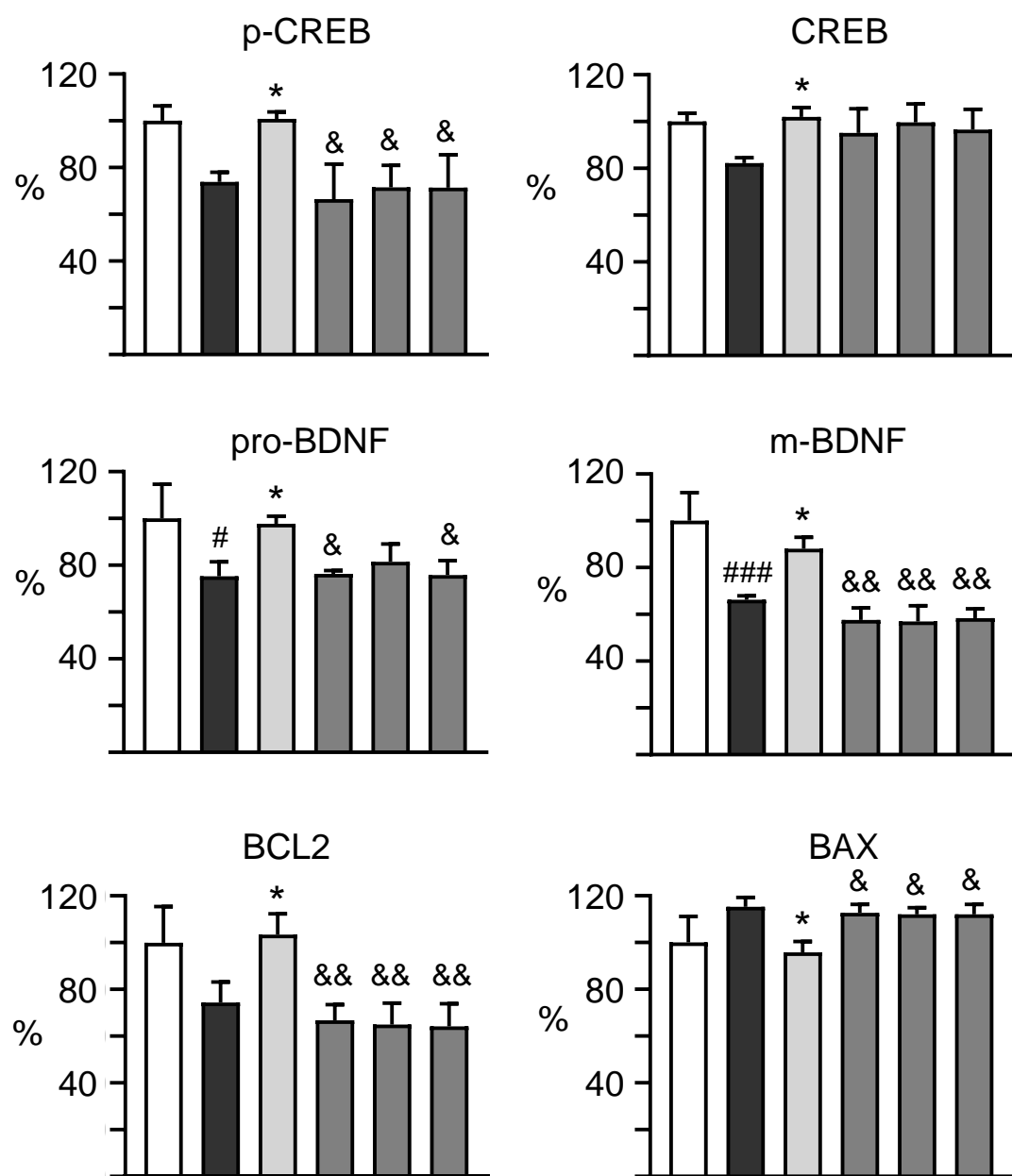

**B**

TUBB3/DAPI

Uninduced

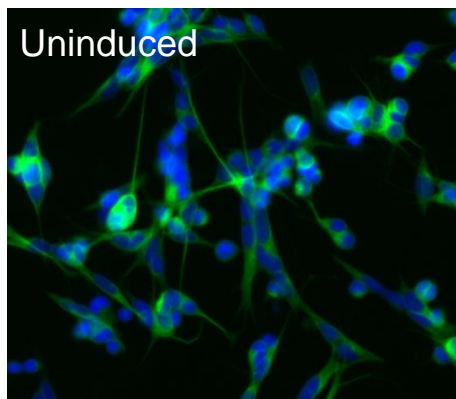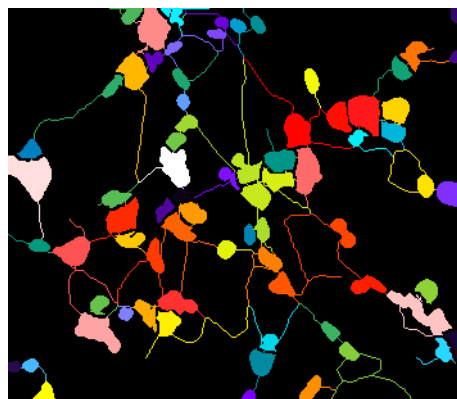

Induced

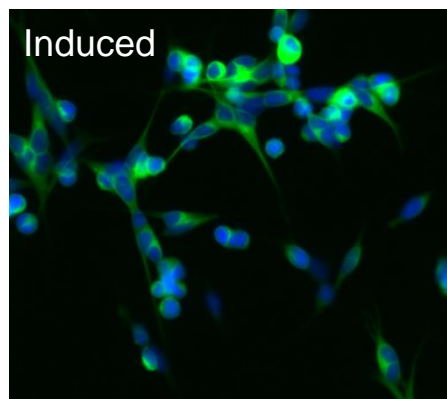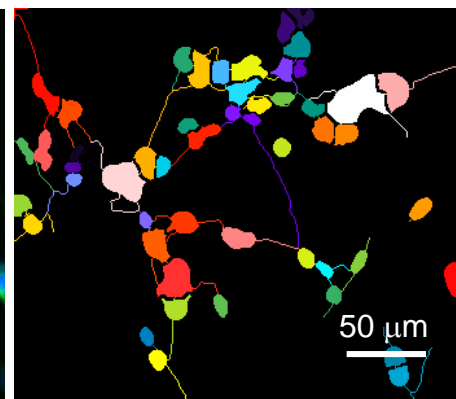

+ LM-021

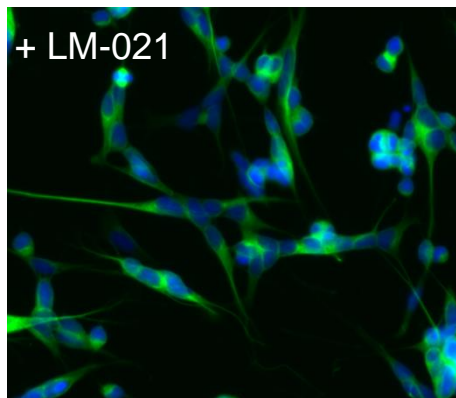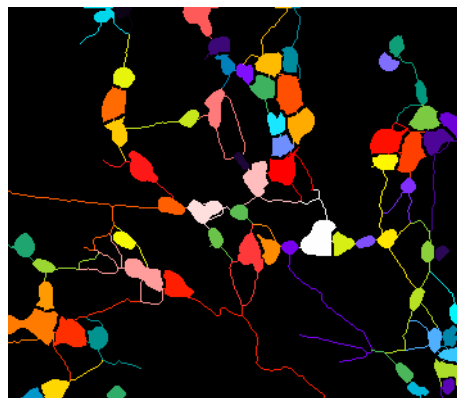

+ LM-021/H-89

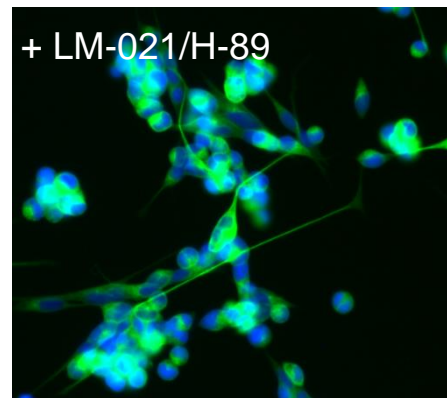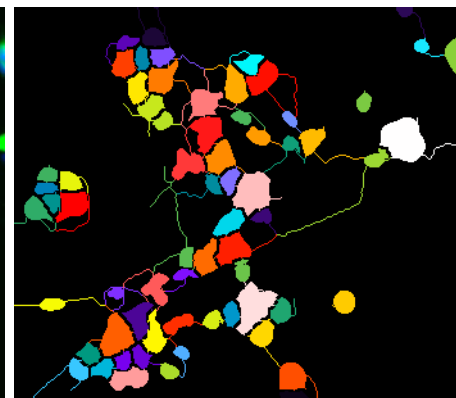

+ LM-021/KN-62

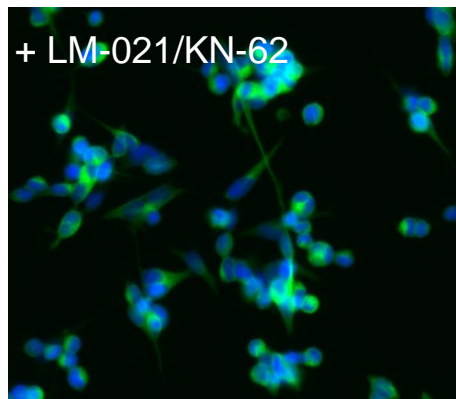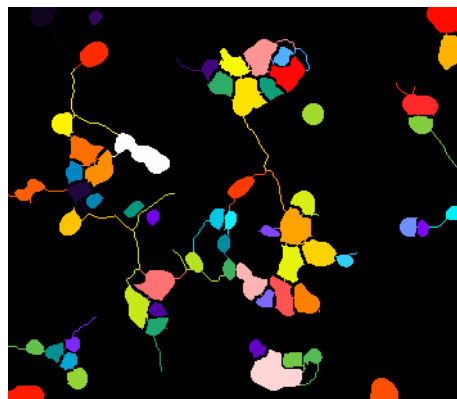

+ LM-021/U0126

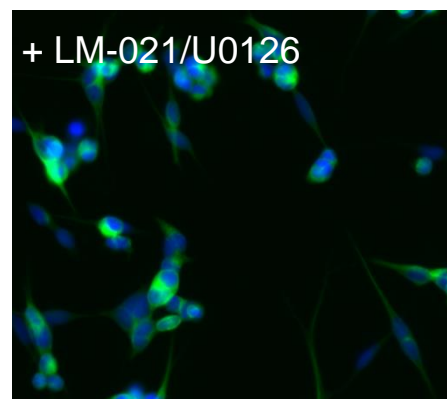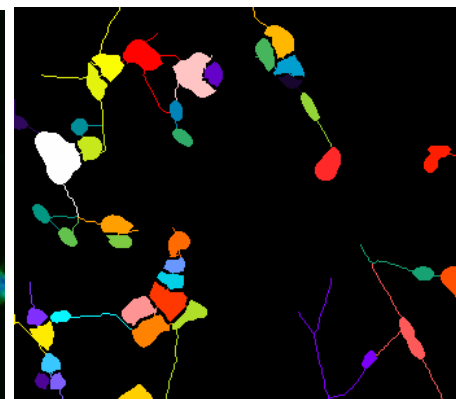

Supplement: Supplementary Materials — Preparation and analysis of mouse plasma and brain homogenate samples. Figure S1: oral bioavailability prediction and TEM examination of Aβ and tau aggregates. Figure S2: nucleotide sequence of synthetic CRE fused to TATA-like promoter and CRE fluorescence reporter assay. Figure S3: dose-response curves based on GFP fluorescence and ROS images in Aβ-GFP cells. Figure S4: dose-response curves based on DsRed fluorescence and ROS images in ΔK280 tauRD-DsRed cells. Figure S5: experimental flow chart to examine LM-021-mediated kinase activation. Figure S6: regulation of CREB signaling pathway and neurite outgrowth images in Aβ-GFP cells. Figure S7: regulation of CREB signaling pathway and neurite outgrowth images in ΔK280 tauRD-DsRed cells. [file 3058861.f1.zip › 3058861.f1/Fig-S7.pdf]
